# Upregulation of SQSTM1 Regulates Ferroptosis and Oxidative Stress in Müller Cells of the Diabetic Neural Retina by Modulating ACSL4

**DOI:** 10.1155/jdr/1924668

**Published:** 2025-08-13

**Authors:** Xinlu Li, Bai Li, Defei Feng, Han Hu, Binyang Tang, Jingying Yang, Huaiyan Jiang, Li Li, Xiaojing Dong, Ninghua Ni, Yan Mei

**Affiliations:** ^1^Faculty of Life Science and Technology, Kunming University of Science and Technology, Kunming, China; ^2^Department of Ophthalmology, The Affiliated Hospital of Kunming University of Science and Technology, Kunming, China; ^3^Department of Ophthalmology, The First People's Hospital of Yunnan Province, Kunming, China; ^4^Medical School, Kunming University of Science and Technology, Kunming, China; ^5^Center for Clinical Medicine Research, The First People's Hospital of Yunnan Province, Kunming, China; ^6^Affiliated Dehong People's Hospital of Kunming Medical University, Dehong, Yunnan, China; ^7^Changsha Aier Eye Hospital, Changsha, China; ^8^The First Affiliated Hospital of Chongqing Medical University, Chongqing, China

**Keywords:** diabetic neuropathy in retina (DNR), ferroptosis, Müller cell, oxidative stress, SQSTM1

## Abstract

Diabetic retinopathy (DR), a leading cause of vision impairment worldwide, is characterized by early neuronal damage in the retina, termed diabetic neuropathy in the retina (DNR). This condition is marked by neuronal apoptosis and glial activation. Müller glia are retinal cells highly susceptible to diabetic metabolic stress that may undergo ferroptosis, an iron-dependent form of regulated cell death driven by lipid peroxidation. However, the role of ferroptosis in DNR pathogenesis remains undefined. In this study, we investigated Müller cell injury under high-glucose and palmitic acid (HGP) conditions. The retinal tissues were obtained from normal rabbits and alloxan-induced diabetic rabbits. HGP exposure significantly reduced Müller cell viability, induced cell cycle arrest, and elevated proinflammatory cytokines. Ultrastructural analysis revealed mitochondrial damage, accompanied by decreased glutathione (GSH) and increased malondialdehyde (MDA), ferrous iron (Fe^2+^), and reactive oxygen species (ROS) levels. RNA sequencing (RNA-Seq) identified *SQSTM1* as a ferroptosis-related differentially expressed gene, which was significantly upregulated in HGP-treated cells. In vivo, DNR rabbits exhibited oxidative stress, iron dysregulation, and elevated SQSTM1 expression that colocalized with GFAP^+^ Müller cells. Single-cell RNA-Seq of human proliferative diabetic retinopathy (PDR) retinas confirmed elevated SQSTM1 expression in Müller cells compared to healthy control (HC) retinas. Mechanistically, *SQSTM1* knockdown attenuated ferroptosis, oxidative stress, and HGP-induced injury, while its overexpression exacerbated ferroptosis via ACSL4 upregulation. Overall, our findings suggest that SQSTM1 may serve as a critical mediator linking Müller cell dysfunction and ferroptosis in DNR pathogenesis, offering a novel potential therapeutic target.

## 1. Introduction

Diabetic retinopathy (DR), the most common neurovascular complication of diabetes, is a leading cause of vision loss in working-age adults due to blood–retina barrier breakdown, neurodegeneration, glial dysfunction, and other pathogenic processes [1, 2]. In the early stages of DR, specifically diabetic neuropathy in the retina (DNR), it is characterized by neuronal apoptosis and glial activation [3]. The Müller glial cells are among the last cells generated during development and are essential for maintaining retinal homeostasis and structural integrity [4]. However, given the limited effectiveness of current treatments in reversing vision loss, elucidating the pathogenesis of DNR is of paramount importance.

Ferroptosis is a regulated cell death mode first reported in 2012, characterized by iron-dependent lipid peroxide accumulation that disrupts membrane integrity [5–7]. Ferroptosis may contribute to the pathogenesis of DR by promoting retinal cell death, including retinal capillary endothelial and glial cells [8, 9]. Current evidence suggests that in DR, retinal iron accumulation drives the generation of oxidizing free radicals and peroxides, which promote lipid peroxidation and impair the function of ferroptosis-regulating proteins [10]. The unique catalytic activity of glutathione (GSH) peroxidase 4 (GPX4) involves the reduction of phospholipid hydroperoxides (PLOOH) to their corresponding alcohols. This activity, facilitated by upstream cyst(e)ine uptake and GSH biosynthesis, constitutes the primary mechanism for ferroptosis suppression [11]. The cystine/glutamate antiporter SLC7A11 (xCT) further protects cells by importing extracellular cystine, thereby elevating intracellular GSH levels and enabling efficient GPX4-mediated detoxification of lipid peroxides [12]. Interestingly, ferroptosis triggered by inhibition of SLC7A11 or GPX4 can be largely abrogated through inactivation of acyl-CoA synthetase long-chain family member 4 (ACSL4) [13]. ACSL4 catalyzes the formation of arachidonoyl-CoA from long-chain fatty acids, priming membrane phospholipids for peroxidation and thus driving the execution of the ferroptotic program [10]. Although resveratrol has been shown to counteract ferroptosis in Müller cells during early DR via the Nrf2/GPx4/PTGS2 pathway [14], and lipid peroxidation is present in diabetic retinas [3, 15, 16], its direct role in Müller cell injury and the underlying mechanisms remain unclear.

Moreover, aberrant expression of SQSTM1/p62 has been closely associated with the development of various neurodegenerative diseases, tumors, infectious and genetic disorders, and chronic conditions [17]. It has been reported that *Xiao Jianzhong Tang (XJZ)* mitigates aspirin-induced oxidative stress and ferroptosis and subsequent gastric mucosal injury by modulating the p62/Keap1/Nrf2 pathway [18], whereas TRIM21 exacerbates these processes via the SQSTM1-NRF2-KEAP1 axis [19]. However, no studies have hitherto addressed the role of SQSTM1 in regulating ferroptosis in DNR.

Over the past few years, ferroptosis has been implicated in retinal diseases, yet its direct versus indirect effects remain unclear. This study directly explored the ferroptosis phenomenon in DNR and explored the underlying regulatory mechanisms. In conjunction with previous studies [20], these findings not only broaden our understanding of the interplay between Müller cells and ferroptosis but also provide new potential targets for the early diagnosis and treatment of DNR and related diseases.

## 2. Materials and Methods

### 2.1. Cell Culture and Establishment of DNR Cell Model

Primary mouse Müller cells (MIC-iCELL-m018; iCell Bioscience Inc., Shanghai) were maintained in Gibco DMEM/F12 medium (Thermo Fisher Scientific) supplemented with 10% fetal bovine serum and 1% penicillin–streptomycin. To confirm Müller cell identity and purity, primary cells were subjected to immunofluorescence (IF) staining using Müller-specific markers glutamine synthetase (GS) and vimentin [21, 22]. Over 90% of the harvested cells stained positive for both markers, demonstrating high purity of the preparation (Figure S1). Cells between Passages 3 and 4 were utilized for experiments. For DNR induction, cells were cultured in DMEM/F12 medium containing 25 mM high-glucose D-glucose and 200 *μ*M palmitic acid (HGP) for 24 h.

### 2.2. Cell Transfection

The sh-SQSTM1, pc-SQSTM1, sh-NC, and pc-NC plasmids were synthesized by Hanbio (Shanghai, China). Müller cells were transfected with these plasmids using Lipofectamine 2000 (Thermo Fisher Scientific, United States) according to the manufacturer's protocol.

### 2.3. Reverse Transcription Quantitative Polymerase Chain Reaction (RT-qPCR)

Total RNA was extracted from tissues and cells using TRIzol reagent (TaKaRa, Japan) and reverse transcribed into cDNA with a reverse transcription kit (TaKaRa, Japan). For amplification, 3 *μ*L of the cDNA reaction product was used. The PCR conditions were as follows: 95°C for 10 min, followed by 35 cycles of 95°C for 12 s, 65°C for 10 s, and 72°C for 20 s. The relative expression levels of GPX4, xCT, and Sqstm1 mRNA were calculated using the 2^−ΔΔCt^ method, with GAPDH as the internal control (Table S1).

### 2.4. Western Blotting

Total proteins were extracted from cells and tissues using RIPA lysis buffer (ServiceBio, Wuhan, China). After determining protein concentrations, 30 *μ*g of protein was loaded onto sodium dodecyl sulfate-polyacrylamide gel electrophoresis for separation. The proteins were then transferred onto polyvinylidene fluoride membranes, which were blocked with 5% skim milk for 1.5 h at room temperature. The membranes were incubated overnight with primary antibodies: for rabbit retinal tissue, anti-xCT (TD12509, Abmart, China), anti-GPX4 (AF7020, Beyotime Biotechnology, China), and anti-Sqstm1 (TA5384, Abmart, China); for mouse cells, anti-xCT (AB307601, Abcam, United States), anti-GPX4 (#52455, Cell Signaling Technology, United States), and anti-Sqstm1 (#5114S, Cell Signaling Technology, United States). Following primary antibody incubation, membranes were incubated with a secondary antibody (ZenBio, China). Protein bands were detected using an enhanced chemiluminescence kit (Millipore Sigma, United States), and protein expression was analyzed and quantified using ImageJ software.

### 2.5. Cell Count Kit-8 (CCK-8) Assay

Cell proliferation was assessed using the CCK-8 kit (Solarbio, Beijing, China). The Müller cells were seeded into 96-well plates at a density of 6 × 10^3^ cells per well and cultured for 48 h. Ten microliters of CCK-8 reagent was added to each well, followed by a 2-h incubation. The optical density (OD) was measured at 450 nm using a microplate reader.

### 2.6. Cell Cycle

Cell cycle analysis was performed using a cell cycle kit (Elabscience). Cells were collected, washed with phosphate-buffered saline, and fixed in 70% ethanol for 3 h at room temperature. After fixation, the cells were rinsed with PBS, centrifuged, and incubated with RNase A at 37°C for 30 min. Propidium iodide dye solution was then added to the cells in the dark for 20 min. Cell cycle distribution was analyzed using flow cytometry.

### 2.7. Reactive Oxygen Species (ROS) Measurement

DCFH-DA (Beyotime Biotechnology) was diluted in serum-free culture medium at a 1:1000 ratio to achieve a final concentration of 10 *μ*M and stored at −20°C protected from light. Müller cells were seeded in 6-well plates at a density of 1 × 10^5^ cells per well and processed according to the experimental protocol. After removing the culture medium, the cells were washed twice with 1× PBS, and the residual medium was aspirated. An appropriate volume (at least 1 mL per well) of diluted DCFH-DA was added to cover the cells, and the plates were incubated for 20 min at 37°C. The cells were then washed three times with serum-free medium to remove unincorporated DCFH-DA. For the ROS positive control, only designated wells were treated with Rosup, which typically induces a significant increase in ROS levels after 20–30 min of stimulation. Finally, samples were examined directly using laser confocal microscopy (Zeiss LSM880).

### 2.8. GSH, Malondialdehyde (MDA), and Ferrous Iron (Fe^2+^) Measurement

Cellular and tissue levels of GSH, MDA, and Fe^2+^ were quantified using detection kits (Solarbio for GSH and MDA; Biosharp for Fe^2+^). Cell supernatants and tissue homogenates were prepared per the manufacturer' instructions, and absorbance was measured using a microplate reader.

### 2.9. Experimental Animals and Establishment of the DNR Model

The study protocol was approved by the Animal Ethics Committee of Kunming Medical University, Yunnan Province, China, in accordance with the Use of Animals in Ophthalmic and Vision Research guidelines (Ethical Approval No.: KMMU20221504). Male New Zealand white rabbits (2.0–3.0 kg; *n* = 3/group) were housed at 21°C (12 h light/dark, 60% humidity) with ad libitum feed. After 12 h fasting, rabbits received IV alloxan (80 mg/kg) or saline. Anesthesia was induced with xylazine (5 mg/kg, IM) followed by ketamine (80 mg/kg, IV). Blood glucose was measured every 6 h for 1 week using an Accu-Chek meter (Roche Diabetes Care, Indianapolis, United States); hyperglycemia was defined as > 16.7 mmol/L (300 mg/dL) on two occasions [23].

### 2.10. Hematoxylin and Eosin (H&E) Staining

Rabbit eyes were fixed in 4% buffered paraformaldehyde at room temperature for 24 h, embedded in paraffin, sectioned, and stained with H&E according to the manufacture's protocol. Sections were then examined by light microscopy.

### 2.11. Transmission Electron Microscopy (TEM)

The eyes were enucleated and fixed in glutaraldehyde at 4°C overnight. The Müller cell pellets were collected by centrifugation, washed in phosphate buffer, and postfixed with osmium tetroxide for 2 h. After a 20-min incubation in 3% EDTA, a 2 × 2-mm piece of eye-cup tissue was excised from the perioptic disc region, identified via multimodal imaging to target areas with the highest density of drusen-like lesions. The tissue was then postfixed in 1% osmium tetroxide in 0.1 M cacodylate buffer for 2 h at 4°C. Following dehydration in a graded acetone series, the tissue was embedded in epoxy resin (Epon 812), and ultrathin sections (50 nm) were cut using an ultramicrotome (EM UC7, Leica, Germany). The sections were stained with 2% uranyl acetate in water and lead citrate for contrast and examined using a JEM-1400PLUS TEM (JEOL Ltd., Tokyo).

### 2.12. IF Staining

Tissue sections were dewaxed in 100% xylene, rehydrated through a graded ethanol series, and rinsed with PBS. Antigen retrieval was performed by heating the sections in citrate buffer (pH 6; Vector Laboratories) for 20 min, followed by a 20-min cooling period at room temperature. Nonspecific binding was blocked by incubating the sections for 1 h at room temperature with a blocking buffer containing 0.01% Triton X-100 (Sigma-Aldrich, T8787-100ML) and 10% donkey serum (Jackson ImmunoResearch, 017-000-121) or goat serum (Sigma-Aldrich, G9023-10ML). The sections were then incubated overnight at 4°C with primary antibodies against GFAP (GB11096-50; Servicebio), xCT (TD12509; Abmart, China), GPX4 (AF7020; Beyotime Biotechnology, China), and Sqstm1 (bs-2951R; Bioss, China). After washing, the sections were incubated with the appropriate secondary antibodies for 2 h at room temperature, and cell nuclei were counterstained with DAPI (Servicebio, G1012-100ML) for visualization.

### 2.13. RNA Sequencing (RNA-Seq) and Gene Set Enrichment Analysis (GSEA)

Following HGP treatment, total RNA was extracted and sequenced using the Illumina HiSeq 2000 platform by BGI Tech (Shenzhen, China), and gene expression was quantified as reads per kilobase of transcript per million mapped reads [24].

### 2.14. Data Sources

Gene expression microarray profiles were retrieved from the NCBI Gene Expression Omnibus (GEO) (http://www.ncbi.nlm.nih.gov/geo) under accession numbers GSE201333 (*n* = 3 healthy controls [HCs]) [25] and GSE165784 (*n* = 6 proliferative diabetic retinopathy [PDR]) [26]. PDR diagnosis was based on the original study criteria, utilizing Early Treatment Diabetic Retinopathy Study grading of fundus photography with supporting OCT findings. Ferroptosis-related gene annotations were obtained from the FerrDb database (http://www.zhounan.org/ferrdb/current/).

### 2.15. Data Preprocessing and Identification of Differentially Expressed Genes (DEGs)

Array data were normalized using the normalizeBetweenArrays function from the limma package, followed by batch-effect correction with ComBat (sva). DEGs (adjusted *p* < 0.05, |log₂FC| > 0.5) were identified using limma, and heatmaps and volcano plots were generated with pheatmap and ggplot2.

### 2.16. Functional Enrichment Analysis

Gene Ontology (GO) and Kyoto Encyclopedia of Genes and Genomes (KEGG) analyses were performed using the R “clusterProfiler” package to identify common DEGs across datasets. These analyses revealed potential biological functions and signaling pathways associated with PDR. Dot plots were used to display the Top 20 GO terms, including biological processes, cellular components, and molecular functions, while bar plots illustrated the top 30 KEGG pathways. Statistical significance was set at a *p* value < 0.05.

### 2.17. Single-Cell Analysis

Low-quality cells, defined as those with a unique molecular identifier (UMI) count below 200 or above 2500 and cells with more than 20% mitochondrial content, were excluded. Gene expression data were normalized using the “NormalizeData” function and scaled with the “ScaleData” function. Batch effects were addressed by integrating single-cell data using the Harmony package. Clustering results were visualized using uniform manifold approximation and projection (UMAP). Finally, single-cell RNA sequencing (scRNA-seq) data were annotated using the “SingleR” R package.

### 2.18. Statistical Analysis

Data were expressed as mean ± SD. Two-group comparisons employed unpaired *t*-tests; multiple groups were compared by one-way ANOVA with Tukey's post hoc test. Analyses were conducted in GraphPad Prism 10. A *p* value < 0.05 was considered statistically significant.

## 3. Results

### 3.1. High Glucose Damages Müller Cells, Inducing Ferroptosis and Oxidative Stress

**Figure 1:**
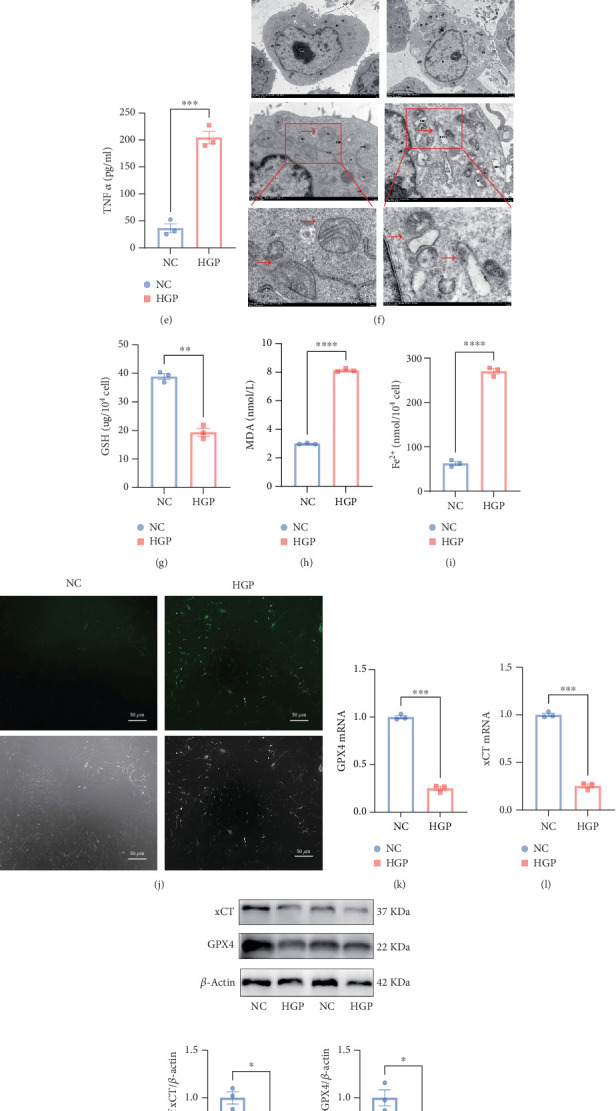
HGP-induced ferroptosis and mitochondrial damage in Müller cells. (a) CCK-8 of Müller cells. (b) Cell cycle analysis was conducted by flow cytometry. (c–e) IL-1*β*, IL-6, and TNF-*α* by ELISA. (f) TEM revealed mitochondrial shrinkage and cristae loss in HGP-treated cells (red arrows). (g–i) Quantification of intracellular GSH, MDA, and Fe^2+^ levels. (j) ROS level was analyzed by fluorescence imaging. (k, l) qPCR analysis revealed GPX4 and xCT mRNA expression. (m) Western blot analysis showed xCT and GPX4 protein levels. HGP: high glucose + palmitic acid. CCK-8: cell count kit-8; TEM: transmission electron microscopy; GSH: glutathione; MDA: malondialdehyde; Fe^2+^: ferrous iron; ROS: reactive oxygen species. Data are expressed as the mean ± SD (*n* = 3). ∗*p* < 0.05, ∗∗*p* < 0.01, ∗∗∗*p* < 0.001, ∗∗∗∗*p* < 0.0001.

We first conducted CCK-8 assays to assess the effect of HGP concentrations on Müller cell viability [27]. As shown in Figure S2A–C, exposure to 25 mmol/L glucose combined with 200 *μ*mol/L palmitic acid for 24 h resulted in a significant reduction in cell viability compared to control conditions (*p* < 0.001). Further evaluation of Müller cells under optimized HGP conditions revealed pronounced cytotoxic effects, with quantitative analysis confirming a significant decrease in cell viability following HGP treatment (Figure 1a*p* < 0.001). Consistent with these findings, CCK-8 assays and flow cytometry indicated that high glucose exposure suppressed Müller cell proliferation (Figure 1b) and induced cell cycle arrest at the G0/G1 phase, preventing progression to the S phase. Besides, HGP-treated cells exhibited elevated levels of proinflammatory cytokines, including IL-1*β* (Figure 1c), IL-6 (Figure 1d), and TNF-*α* (Figure 1e).

TEM of HGP-treated cells revealed characteristic morphological alterations, including irregular nuclear contours with pronounced membrane indentation, perinuclear space widening, and chromatin redistribution, manifesting as reduced heterochromatin. Mitochondrial abnormalities, including swelling, cristae disintegration, and loss of electron density, were also observed (Figure 1f).

Biochemical analysis showed significant depletion of GSH levels (Figure 1g), elevated MDA content (Figure 1h), and increased Fe^2+^ accumulation (Figure 1i). Fluorometric analysis revealed an increase in ROS production (Figure 1j). Molecular analysis via qPCR and Western blot demonstrated downregulation of key ferroptosis regulators, including GPX4 (Figures 1k, 1l, and 1m) and xCT (Figure 1l,m).

These findings collectively indicated that HGP exposure could induce ferroptosis in Müller cells via potentiation of redox imbalance and activation of lipid peroxidation pathways.

### 3.2. Identification of Hub DEGs Related to Ferroptosis and Müller Cells

**Figure 2:**
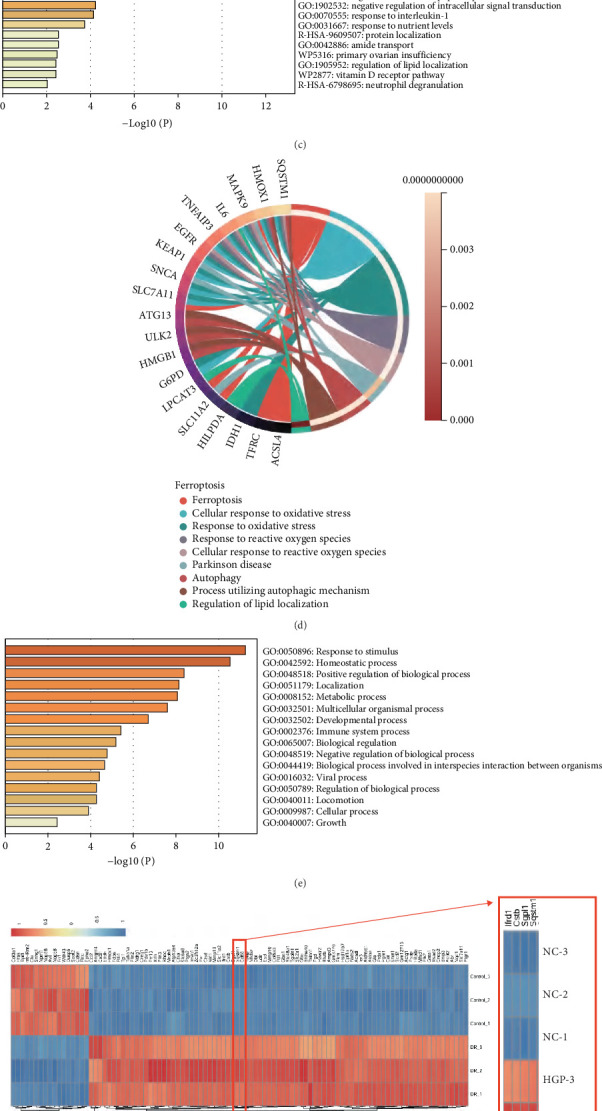
RNA-seq analysis identified SQSTM1 as a ferroptosis-related gene of Müller cells. (a) RNA-seq analysis revealed 3214 DEGs. (b) Venn diagram illustrating 49 ferroptosis-related DEGs, including 25 upregulated and 13 downregulated genes. (c) GO enrichment analysis indicated involvement in stimulus response, immune regulation, homeostasis, and metabolism. (d) Gene–GO term interaction network revealed functional synergy among ferroptosis-related genes. (e) KEGG pathway analysis showed enrichment in ferroptosis, oxidative stress, hypoxia adaptation, apoptosis, autophagy, and metabolic pathways. (f) Heatmap highlighted significant upregulation of SQSTM1 in the HGP group. (g–n) qPCR analyses confirmed upregulation of Per, Ptgs2, Nqol, Slc2al, Gla, xCT, Cat, and SQSTM1 under high-glucose + palmitic acid conditions.(o) Western blot analyses confirmed upregulation of SQSTM1 under HGP conditions. Data are expressed as the mean ± SD (n = 3). DEGs: differentially expressed genes; HGP: high glucose + palmitic acid; GO: Gene Ontology; KEGG: Kyoto Encyclopedia of Genes and Genomes; ∗*p* < 0.05, ∗∗*p* < 0.01, ∗∗∗*p* < 0.001, ∗∗∗∗*p* < 0.0001.

The mechanism by which ferroptosis-mediated Müller cell death contributes to DNR remains unclear. In this study, RNA-seq analysis of mouse retinal Müller cells treated with HGP for 24 h identified 3214 DEGs, with 1734 genes upregulated and 1480 downregulated in the HGP group (Figure 2a). The intersection of these DEGs with ferroptosis-related genes (*n* = 264) yielded 49 DEGs closely associated with ferroptosis (upregulated: *n* = 25 and downregulated: *n* = 14, Figure 2b), with SQSTM1 exhibiting significant upregulation in the HGP group.

To further elucidate the functional roles of these ferroptosis-related DEGs, GO and KEGG enrichment analyses were performed. GO analysis revealed significant enrichment in biological processes such as response to stimuli, immune system processes, homeostasis, and metabolic processes (Figure 2c). Gene–GO term interaction analysis (Figure 2d) suggested a synergistic effect among these genes, indicating a complex regulatory mechanism underlying ferroptosis. KEGG analysis showed that these genes were primarily involved in pathways related to ferroptosis, oxidative stress response, adaptation to hypoxia, apoptosis, and autophagy, as well as several immune and metabolic signaling pathways (Figure 2e), highlighting the multifaceted regulation of ferroptosis under high-glucose conditions.

Further analysis of gene–GO term interactions revealed a strong association between *SQSTM1* and ferroptosis. Among the 25 upregulated genes, a review of the literature revealed that eight genes (*Per*, *Ptgs2*, *Nqol*, *Slc2al*, *Gla*, *xCT*, *Cat*, and *SQSTM1*) have been reported in DR studies (Figures 2g, 2h, 2i, 2j, 2k, 2l, 2m, and 2n). The qPCR validation of these candidates revealed consistent expression patterns for seven genes (*Per*, *Ptgs2*, *Nqol*, *Slc2al*, *Gla*, *xCT*, and *SQSTM1*) with the microarray heatmap results. Subsequent review of existing literature on ferroptosis in DR indicated limited investigation of SQSTM1's role in this context. Western blot analyses (Figure 2o) demonstrated significant upregulation of SQSTM1 expression under high-glucose conditions (*p* < 0.01), correlating with transcriptional-level results. These findings collectively underscore the pivotal role of *SQSTM1* as a regulatory gene in high-glucose-induced ferroptosis in Müller cells.

### 3.3. Ferroptosis and Oxidative Stress Activation With SQSTM1 Upregulation in DNR Rabbits

**Figure 3:**
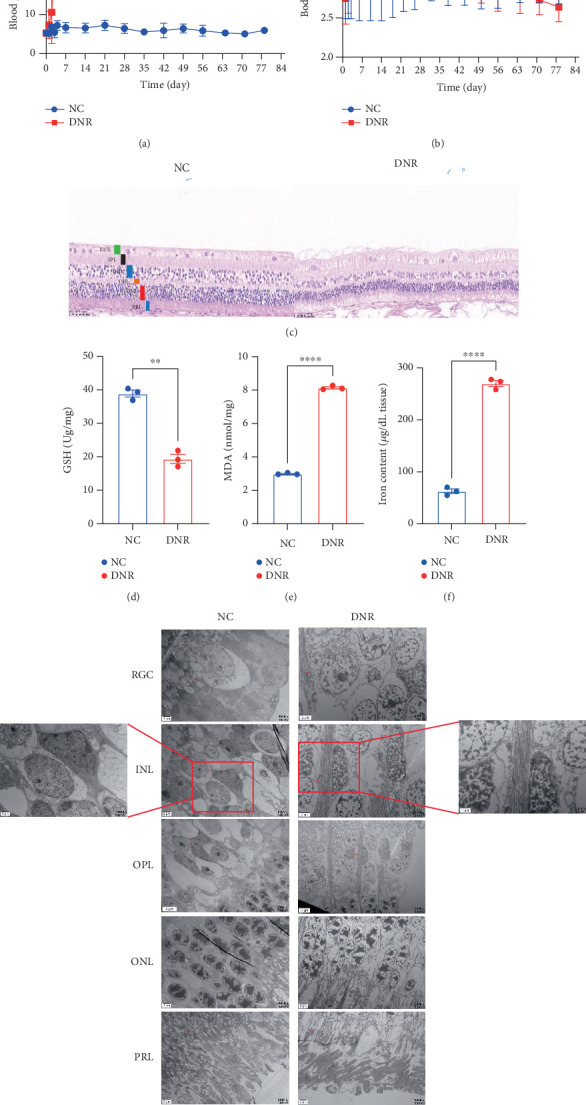
DNR rabbits induced hyperglycemia, weight loss, retinal thinning, and ferroptosis increased in rabbits. (a) DNR rabbits exhibited significantly elevated fasting blood glucose. (b) DNR rabbits experienced significant body weight loss. (c) Representative H&E results. (d–f) Quantification of intracellular GSH, MDA, and Fe^2+^ levels. (g) TEM revealed mitochondrial swelling and cristae loss in the DNR retina. Data are expressed as the mean ± SD (*n* = 3). DNR: diabetic neuropathy in the retina; NC: normal control; H&E: hematoxylin and eosin; TEM: transmission electron microscopy; GSH: glutathione; MDA: malondialdehyde; Fe^2+^: ferrous iron; ROS: reactive oxygen species; GCL: ganglion cell layer; IPL: inner plexiform layer; INL: inner nuclear layer; OPL: outer plexiform layer; ONL: outer nuclear layer; PRL: photoreceptor layer; RGC: retinal ganglion cells. Data are expressed as the mean ± SD (*n* = 3). ∗*p* < 0.05, ∗∗*p* < 0.01, ∗∗∗*p* < 0.001, ∗∗∗∗*p* < 0.0001.

We next assessed fasting blood glucose levels and body weight changes in the DNR and NC groups. The results showed that blood glucose levels in the DNR group rapidly increased, stabilizing at approximately 18 mmol/L by Day 7, which was significantly higher than in the NC group (Figure 3a). During the experimental period, the NC group demonstrated typical weight gain patterns, whereas the DNR group exhibited progressive weight loss, declining below 2.0 kg by Day 56 (Figure 3b). Histological examination of retinal architecture (Figure 3c) revealed intact, well-organized laminar structure in the normal control (NC) group. Quantitative assessment of retinal thickness revealed the development of retinopathy in DNR, as evidenced by significant thinning of specific retinal layers, including the inner nuclear layer (INL), inner plexiform layer (IPL), and outer nuclear layer (ONL), confirming diffuse cellular apoptosis.

Biochemical analysis (Figures 3d, 3e, and 3f) showed that, compared to the NC group, the DNR group exhibited significantly lower levels of GSH (*p* < 0.01), while MDA content and iron concentrations were significantly elevated (*p* < 0.0001), indicating enhanced oxidative stress and disrupted iron homeostasis in the retina of DNR animals. TEM (Figure 3g) revealed severe cellular structural abnormalities across various retinal layers in the DNR group, including mitochondrial swelling, cristae disruption, and disorganized organelle arrangement.

**Figure 4:**
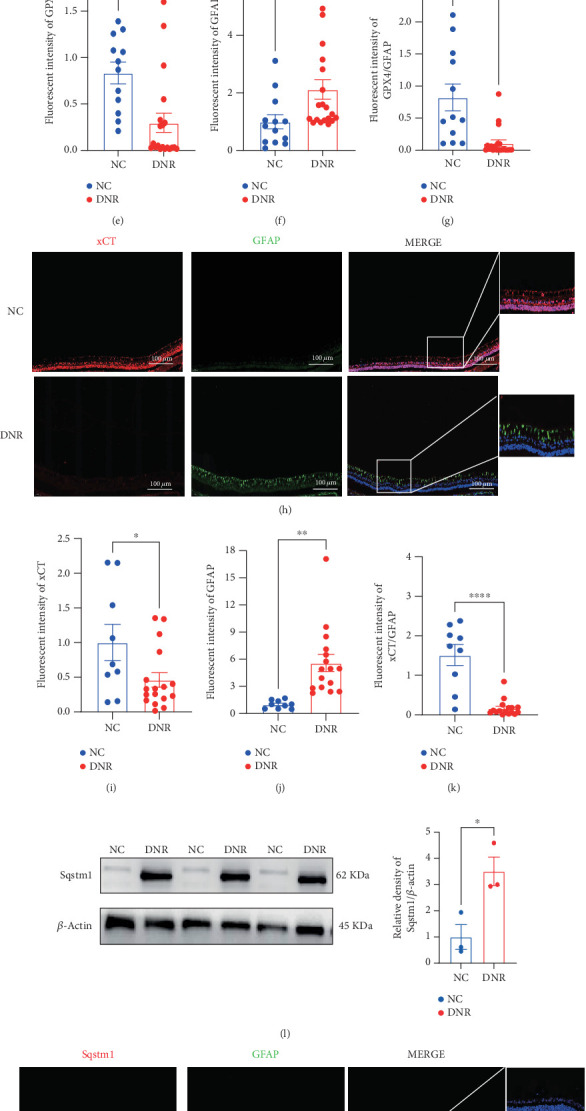
Downregulation of xCT and GPX4 with upregulation of SQSTM1 and glial activation in retinal tissues. (a–c) Western blot analysis showed xCT and GPX4 protein levels. (d–k) Immunofluorescence revealed decreased GPX4 and xCT levels and increased GFAP fluorescence. (l) Western blot analysis showed SQSTM1 protein levels. (m–p) SQSTM1 immunofluorescence demonstrated increased expression and enhanced co-localization with GFAP. Data are expressed as the mean ± SD (*n* = 3). DNR: diabetic neuropathy in the retina; NC: normal control. Data are expressed as the mean ± SD (*n* = 3). ∗*p* < 0.05, ∗∗*p* < 0.01, ∗∗∗*p* < 0.001, ∗∗∗∗*p* < 0.0001.

Western blot analysis showed significantly reduced expression of xCT and GPX4 in the DNR group compared to the NC group (*p* < 0.001) (Figures 4a, 4b, and 4c), while SQSTM1 expression was markedly increased (Figure 4l). IF staining further revealed that in the NC group, GPX4 and xCT were highly expressed with low GFAP fluorescence intensity. In contrast, in the DNR group, GPX4 and xCT were significantly downregulated, and GFAP fluorescence intensity was notably elevated (Figures 4d, 4e, 4f, 4g, 4h, 4i, 4j, and 4k). Besides, while SQSTM1 exhibited a uniform distribution in the NC group, its expression was significantly upregulated in the DNR group, with increased colocalization with GFAP (Figures 4m, 4n, 4o, and 4p). These findings collectively suggest that under hyperglycemic conditions, the downregulation of GPX4 and xCT in the retina is accompanied by glial cell activation, and *SQSTM1* upregulation plays a critical role in activating the ferroptosis pathway in retinal astrocytes and Müller cells.

### 3.4. Single-Cell Transcriptomic Profiling Reveals Müller Cell Heterogeneity and Elevated SQSTM1 Expression in PDR

**Figure 5:**
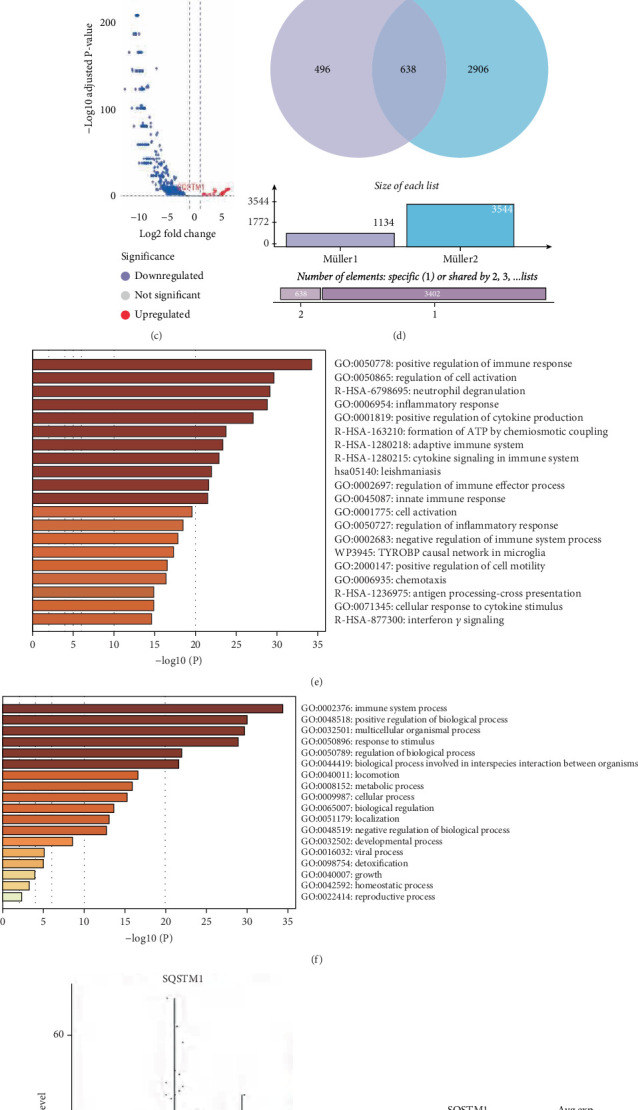
scRNA-seq revealed SQSTM1 upregulation and Müller cell heterogeneity in PDR. (a) UMAP plot identified major retinal cell types, including two Müller subsets. (b) Cell distribution by sample origin (PDR: red; HC: blue) indicated disease-associated shifts. (c) Volcano plot showing distinct gene expression between Müller Cell 1 and 2. (d) Venn diagram depicting shared and unique DEGs between the subsets. (e) GO enrichment highlights oxidative stress, metal ion homeostasis, and autophagy. (f) KEGG analysis implicated iron metabolism, autophagy, and NLRP3 inflammasome pathways. (g) SQSTM1 expression was elevated in PDR retinas. (h) SQSTM1 was primarily upregulated in Müller cells, with increases in other retinal cell types. Single-cell RNA sequencing: scRNA-seq; RGC: retinal ganglion cell; PDR: proliferative diabetic retinopathy; HC: healthy control; GO: Gene Ontology; KEGG: Kyoto Encyclopedia of Genes and Genomes; ∗*p* < 0.05, ∗∗*p* < 0.01, ∗∗∗*p* < 0.001, ∗∗∗∗*p* < 0.0001.

To investigate the expression profile of *SQSTM1* in human retinal cells, we performed scRNA-seq on retinal tissues from patients with PDR and HC.  Figure 5a presents the UMAP clustering of retinal cells into distinct subpopulations, including microglia, bipolar cells, Müller Cell 1, rod cells, pericytes, vascular cells, Müller Cell 2, and retinal ganglion cell (RGC).  Figure 5b shows the distribution of cells by sample origin, revealing spatial segregation between the PDR and HC groups, suggesting that disease conditions induced alterations in cellular states. Differential expression analysis between Müller Cell 1 and Müller Cell 2 identified significant transcriptional differences, visualized in a volcano plot (Figure 5c). A Venn plot (Figure 5d) was next generated to visualize the shared DEGs between the two Müller cell subsets. GO analysis (Figure 5e) revealed significant enrichment in processes related to oxidative stress response, metal ion homeostasis, and autophagy. KEGG analysis (Figure 5f) further identified pathways related to iron homeostasis, autophagy, and NLRP3 inflammasome activation, which are central to diabetic retinal injury. Notably, as shown in Figure 5g, marked upregulation of *SQSTM1* was observed in PDR samples.  Figure 5h revealed that this upregulation was pronounced in the Müller cells, with elevated expression also observed in RGCs, pericytes, rods, bipolar cells, and microglia.

### 3.5. SQSTM1 Promotes Ferroptosis and Oxidative Stress via ACSL4 Upregulation Under High-Glucose Conditions

**Figure 6:**
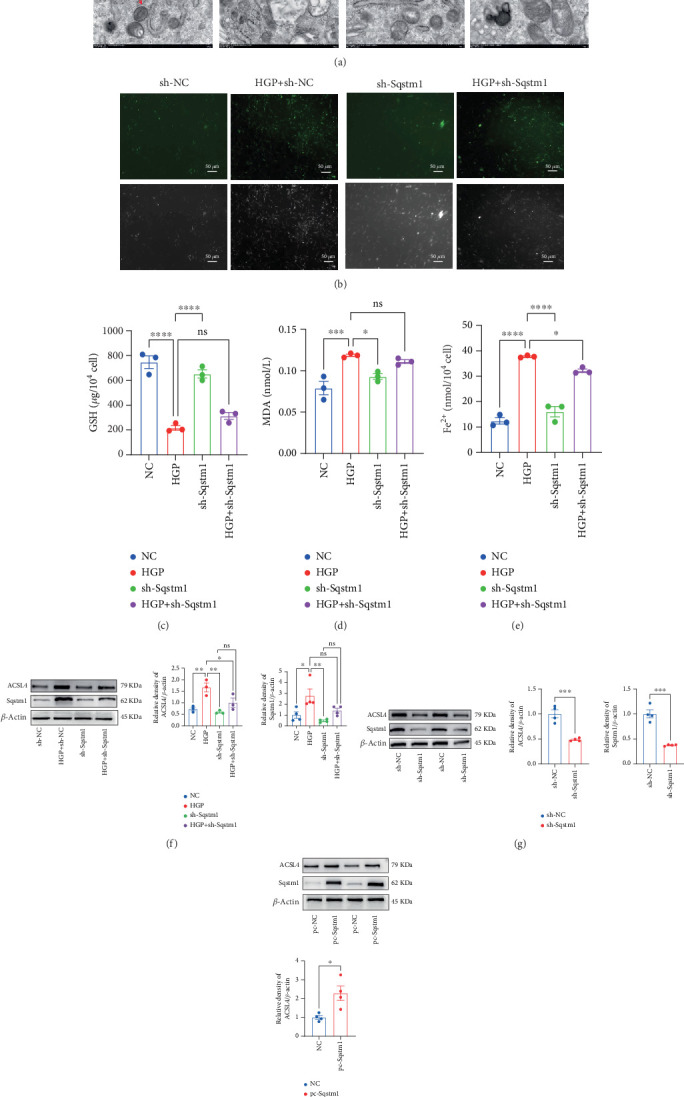
SQSTM1 regulated ferroptosis and oxidative stress in Müller cells by ACSL4. (a) TEM analysis showing mitochondrial damage in HG + sh-NC cells, while HG + sh-SQSTM1 cells exhibited milder alterations and preserved membrane integrity. (b–e) SQSTM1 knockdown reduced ROS, Fe^2+^, and MDA levels, and increased GSH. (f, g) Western blot demonstrating that SQSTM1 knockdown suppressed HGP-induced ACSL4 upregulation. (h) SQSTM1 overexpression enhanced ACSL4 expression. TEM: transmission electron microscopy; GSH: glutathione; MDA: malondialdehyde; Fe^2+^: ferrous iron; ROS: reactive oxygen species. Data are expressed as the mean ± SD (*n* = 3). ∗*p* < 0.05, ∗∗*p* < 0.01, ∗∗∗*p* < 0.001, ∗∗∗∗*p* < 0.0001.

To investigate the dual regulatory function of *SQSTM1* in ferroptosis, we modulated its expression in Müller cells via plasmid transfection. Three sh-SQSTM1 constructs were initially tested, and both immunoblotting and IF analyses identified sh-SQSTM1#2 as the most effective knockdown sequence (Figure S3A,B). The efficiency of *SQSTM1* overexpression was validated (Figure S3C). Under HGP stimulation, *SQSTM1* knockdown significantly attenuated oxidative stress and lipid peroxidation. TEM revealed severe mitochondrial damage, including swelling, cristae disruption, and membrane rupture, in the HGP + sh-NC group, whereas cells with *SQSTM1* knockdown (HGP + sh-SQSTM1) exhibited milder mitochondrial alterations and preserved membrane integrity (Figure 6a). Biochemical assays further demonstrated that in the HGP + sh-SQSTM1 group, levels of ROS (Figure 6b), MDA (Figure 6d), and Fe^2+^ (Figure 6e) were significantly reduced, while GSH levels were elevated (Figure 6c), indicating attenuation of ferroptosis. Moreover, Western blot analysis revealed that *SQSTM1* knockdown suppressed HGP-induced upregulation of ACSL4 protein (Figure 6f,g), whereas *SQSTM1* overexpression enhanced ACSL4 expression (Figure 6h), suggesting that *SQSTM1* positively regulated ACSL4. Collectively, these findings suggest that SQSTM1 promotes ferroptosis and oxidative stress in the Müller cells under hyperglycemic conditions by upregulating ACSL4, highlighting its potential as a therapeutic target for DNR.

## 4. Discussion

DNR represents an irreversible neurodegenerative condition characterized by progressive visual impairment [28, 29]. Retinal neurodegeneration, which precedes microvascular alterations, serves as an early diagnostic indicator of DNR and can be detected before clinical symptoms emerge [30]. Under hyperglycemic conditions, this neurodegeneration is characterized by excitotoxicity, increased neuronal apoptosis, glial reactivity, microglial activation, shifts in glutamate metabolism, and neurotrophins depletion [31]. Previous studies have shown that under high-glucose conditions, Müller cells secrete increased levels of proinflammatory cytokines and exhibit elevated ROS and lipid peroxidation, which disrupt the blood-retina barrier and damage neurons [32–36]. However, the precise mechanism underlying high-glucose-induced Müller cell injury remains unclear. In this study, we examined the pathogenic role and molecular regulation of ferroptosis in DNR. Ferroptosis represents a novel form of regulated cell death distinguished by iron-dependent lipid peroxidation and distinct from classical apoptotic pathways [37].

In recent years, small-molecule ferroptosis inhibitors, such as Fer-1, have been shown to mitigate retinal neurodegeneration and exhibit neuroprotective effects, underscoring the potential role of ferroptosis in early retinal nerve damage in DR [15, 38, 39]. In our experimental models, the retina of diabetic rabbits and high-glucose-cultured retinal Müller cells exhibited decreased mitochondrial abundance and cristae loss, elevated Fe^2+^ levels, reduced GSH synthesis, and increased MDA accumulation. Retinal Müller cells are now understood to perform essential homeostatic functions, including glutamate clearance, ion buffering, neurotrophic support, and antioxidant defense, which are crucial for maintaining retinal neuronal viability [35]. Ferroptosis-induced Müller cell dysfunction precipitates a cascade of neurotoxic events: extracellular glutamate accumulation, ionic imbalance, and impaired neuroprotective signaling [40]. This creates an excitotoxic and oxidative environment that directly damages photoreceptors, bipolar cells, and ganglion cells [41]. In both streptozotocin-induced diabetic mice and high-glucose-treated Müller cells, PRDX4 deficiency exacerbated retinal neurodegeneration, reactive gliosis, apoptosis, ferroptosis, oxidative stress, and mitochondrial dysfunction, whereas PRDX4 overexpression provided significant protection against these diabetic insults [20, 42]. Enhancing PRDX4 levels may represent a promising therapeutic strategy for delaying DR progression [9]. Besides, the effect of HGP on ferroptosis warrants further investigation, possibly by adjusting intervention conditions or using different retinal cell types.

GO and KEGG analyses revealed that these genes were primarily involved in ferroptosis signaling, ROS metabolism, and the NF-*κ*B inflammatory pathway, suggesting that high glucose-induced oxidative stress and inflammation may exacerbate retinal cell injury through ferroptosis. Notably, SQSTM1, a key regulator of oxidative stress-induced inflammation and ferroptosis, is closely associated with the pathophysiology of DNR. Wei et al. [19] found that TRIM21 promoted oxidative stress and ferroptosis by regulating the SQSTM1-NRF2-KEAP1 axis. Based on the above findings, it can be inferred that *SQSTM1* represents an upstream regulatory factor in Müller cell ferroptosis. Under high-glucose conditions, *SQSTM1* was significantly upregulated in Müller cells. In the Alloxan-induced DNR rabbit model, Western blot and IF assays showed that SQSTM1 protein expression in retinal tissues was markedly elevated, accompanied by an increase in GFAP-positive cells, indicative of glial activation. This finding was corroborated in DR samples, STZ-induced diabetic models, and high-glucose–treated human retinal capillary endothelial cells [43, 44]. Furthermore, analysis of human retinal scRNA-seq data categorized Müller cells into two major subpopulations with distinct gene expression profiles; in PDR, Müller cells, RGCs, and bipolar cells all exhibited high *SQSTM1* expression, which is consistent with our in vivo and in vitro results and underscores its multiple roles in retinal pathology. Notably, despite the marked reduction in RGC density observed under PDR conditions, the remaining RGCs exhibited significant upregulation of SQSTM1. Given its role as an upstream modulator of ferroptosis, the increased expression of *SQSTM1* in these cells is likely indicative of a compensatory autophagic mechanism aimed at mitigating ferroptotic signaling and supporting neuronal survival under conditions of hyperglycemia and oxidative stress [45]. One study [46] demonstrated that oral administration of NGR1 prevented DR in *db/db* mice and high glucose-induced damage in retinal Müller cells by inhibiting apoptosis, inflammation, oxidative stress, and abnormal VEGF/PEDF expression, primarily through a PINK1-dependent enhancement of mitophagy. This finding further highlights the dual function of SQSTM1 in regulating both autophagy and ferroptosis [47], as well as its potential contribution to the resilience of specific RGC subtypes.

*SQSTM1* knockdown experiments demonstrated that reducing *SQSTM1* significantly decreased intracellular ROS, MDA, and Fe^2+^ levels under high-glucose conditions, increased GSH, and improved cellular ultrastructure; conversely, *SQSTM1* overexpression promoted the upregulation of the ferroptosis-related protein ACSL4. Emerging evidence highlights the pathogenic role of ACSL4-mediated ferroptosis across multiple diabetic complications [48]. Recent studies have demonstrated that ACSL4-dependent ferroptosis signaling contributes significantly to disease progression in diabetic cardiomyopathy [49, 50], nephropathy [51], and retinopathy [52]. These findings collectively suggest that ACSL4 represents a critical molecular node linking metabolic dysfunction to ferroptosis-driven tissue damage in diabetes. The present study further reveals SQSTM1's novel role in amplifying high-glucose-induced ferroptosis via ACSL4 induction, extending its known function as a master regulator of stress responses and ferroptotic cell death [6]. The potential mechanism of SQSTM1-mediated regulation of ACSL4 remains unclear. Across multiple models, SQSTM1 demonstrates a parallel rise in ACSL4 mRNA and protein, indicating a transcriptional mechanism. For instance, in pancreatic acinar cells [53], extracellular rSQSTM1 treatment elevated ACSL4 transcripts and protein, likely via AGER-dependent signaling. Similarly, in corneal epithelial cells subjected to dry-eye conditions [54], autophagy impairment–induced SQSTM1 accumulation similarly boosted ACSL4 mRNA and protein, while SQSTM1 knockdown reversed these effects. These findings collectively indicate that SQSTM1 upregulates ACSL4 predominantly by enhancing its gene transcription rather than by posttranscriptional stabilization or direct protein binding. However, in distinct neuronal and wound-healing contexts, SQSTM1 governs ACSL4 abundance exclusively via a posttranslational mechanism, involving protein–protein interaction-mediated autophagic degradation. Specifically, in MN9D dopaminergic neurons, SQSTM1 overexpression drives a marked decrease in ACSL4 protein [55]. Furthermore, in diabetic wound-healing models, hyperglycemia-induced SQSTM1 loss impairs autophagic flux, leading to ACSL4 accumulation and enhanced ferroptosis. Conversely, pharmacologic restoration of SQSTM1 reactivates LC3-dependent autophagy to selectively degrade ACSL4 without affecting its mRNA levels [56].

The present study provides evidence that SQSTM1 acts as an upregulator of ferroptosis in DNR by promoting ACSL4 expression in Müller glia, thereby contributing to disease progression. ScRNA-seq analysis revealed SQSTM1 upregulation across multiple retinal cell types, including Müller cells, RGCs, pericytes, rods, bipolar cells, and microglia. An alternative, cytoprotective interpretation of our findings suggests that the concurrent upregulation of SQSTM1 and ACSL4 in Müller glia represents an adaptive mitophagic program recruited to mitigate high-glucose-induced mitochondrial injury and oxidative stress, rather than a purely proferroptotic signal [57]. In this context, SQSTM1 could function as a scaffold for ubiquitinated, damaged mitochondrial fragments, facilitating their delivery to autophagosomes via its LC3-interacting region [58], thereby curtailing ROS generation and preserving ATP production [59]. In addition, Yang et al. [53] reported that *SQSTM1* could enhance AGER-dependent ACSL4 expression, leading to autophagy-dependent ferroptosis. ACSL4, beyond its well-established role in sensitizing membranes to peroxidation, also participates in phospholipid remodeling, a process essential for autophagosome biogenesis and membrane expansion. Therefore, its induction might enhance the clearance of dysfunctional organelles [60]. Previous studies have also indicated its essential role in regulating oxidative stress and autophagy [61], and factors such as xCT, GPX4, NRF2, p53, and ACSL4 have been identified as key regulators of autophagy and ferroptosis [62] Notably, a recent study [56] demonstrated that ACSL4 downregulation paradoxically promoted ferroptosis in periwound tissues of diabetic rats, correlating with accelerated wound healing. Mechanistically, this effect appears to be mediated through the autophagy-lysosome pathway in keratinocytes, where SQSTM1 downregulation inhibits autophagic flux and consequently modulates ACSL4 protein stability.

The study has several limitations that should be acknowledged. First, as our study was conducted using primary mouse Müller cell cultures and a diabetic rabbit model, it cannot fully capture the complex, multifactorial environment of human DR. Second, while previous work indicated that SQSTM1 could mitigate oxidative stress in DR by promoting autophagy to clear excess ROS and damaged cell components [63], our results showed that in DNR, SQSTM1 upregulation promoted ferroptosis and retinal cell injury. SQSTM1 may differentially regulate autophagy, ferroptosis, and other mechanisms at various stages of DR. Third, due to the limited availability of human retinal tissue samples, we were unable to validate SQSTM1 expression using qPCR and immunohistochemistry. Besides, this study provided only preliminary data on the impact of SQSTM1 on ACSL4 expression; the interaction mechanism between SQSTM1 and ACSL4 requires further elucidation.

## 5. Conclusion

**Figure 7:**
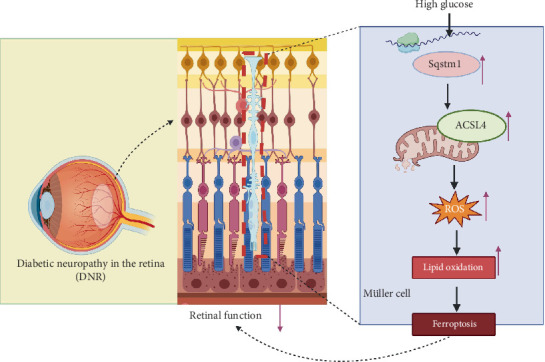
Under high glucose conditions, SQSTM1 upregulation enhanced ACSL4-mediated ferroptosis in Müller cells, thereby promoting diabetic retinal neuropathy progression.

Overall, this study revealed that under high-glucose conditions, the Müller cells could activate ferroptosis through oxidative stress and lipid peroxidation, directly damaging retinal neurons and inducing glial cell activation. *SQSTM1*, acting as an upstream positive regulator of ferroptosis, was upregulated and further exacerbated Müller cell injury by modulating the expression of the ferroptosis-related protein ACSL4 (Figure 7). Overall, our study provided a novel theoretical framework and identified potential therapeutic targets within DNR and the ferroptosis pathway.

## Data Availability

The datasets generated and analyzed during the current study are available from the corresponding authors upon reasonable request.

## Nomenclature

ACSL4: acyl-CoA synthetase long-chain family member 4
DR: diabetic retinopathy
DNR: diabetic neuropathy in the retina
HGP: high-glucose and palmitic acid
GSH: glutathione
MDA: malondialdehyde
Fe^2+^: ferrous iron
ROS: reactive oxygen species
RNA-Seq: RNA sequencing
PDR: proliferative diabetic retinopathy
HC: healthy control
XJZ: Xiao Jianzhong Tang
GS: glutamine synthetase
CCK-8: cell count kit-8
H&E: hematoxylin and eosin
TEM: transmission electron microscopy
IF: immunofluorescence
GSEA: Gene Set Enrichment Analysis
GPX4: glutathione peroxidase 4
DEGs: differentially expressed genes
GO: Gene Ontology
GEO: Gene Expression Omnibus
KEGG: Kyoto Encyclopedia of Genes and Genomes
UMI: unique molecular identifier
UMAP: uniform manifold approximation and projection
NC: normal control
INL: inner nuclear layer
IPL: inner plexiform layer
ONL: outer nuclear layer
OD: optical density
PLOOH: phospholipid hydroperoxides
RGC: retinal ganglion cell
RT-qPCR: reverse transcription quantitative polymerase chain reaction
ScRNA-seq: single-cell RNA sequencing
SLC7A11: Solute Carrier Family 7 Member 11

## Supporting Information

Supporting Information Additional supporting information can be found online in the Supporting Information section. The supporting information for this article can be found online at the following: Figure S1: Immunofluorescence identification of Müller cells. Figure S2: High glucose/palmitic acid (HGP) treatment reduces Müller cell viability. Figure S3: Validation of Sqstm1 knockdown and overexpression efficiency in Müller cells. Table S1: Forward and reverse sequences of each gene analyzed by real-time polymerase chain reaction.
