# Supplementary material for: Upregulation of SQSTM1 Regulates Ferroptosis and Oxidative Stress in Müller Cells of the Diabetic Neural Retina by Modulating ACSL4
Source: J Diabetes Res. 2025 Aug 13;2025:1924668. doi: 10.1155/jdr/1924668 (PMC12367365; doi:10.1155/jdr/1924668)
Supplement: Supporting Information — Additional supporting information can be found online in the Supporting Information section. The supporting information for this article can be found online at the following: Figure S1: Immunofluorescence identification of Müller cells. Figure S2: High glucose/palmitic acid (HGP) treatment reduces Müller cell viability. Figure S3: Validation of Sqstm1 knockdown and overexpression efficiency in Müller cells. Table S1: Forward and reverse sequences of each gene analyzed by real-time polymerase chain reaction. [file 1924668.f1.zip › Supplementary Figure2.pdf]

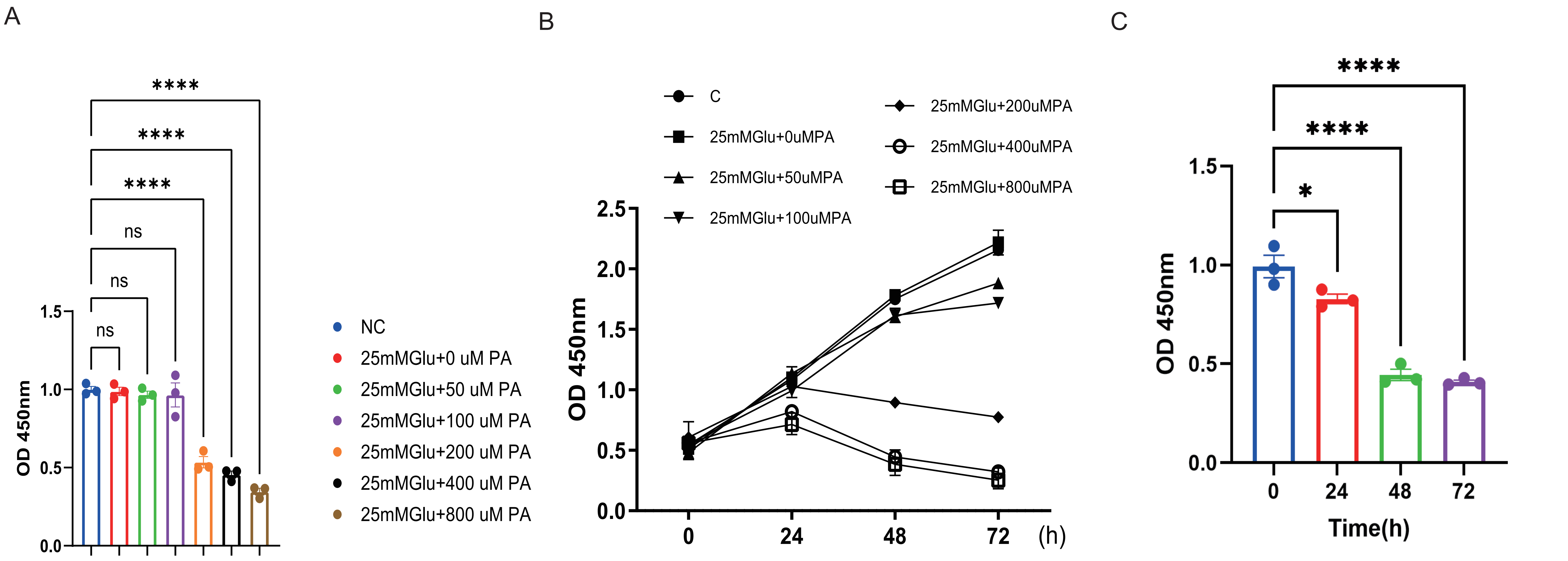
A
B
C
****
****
****
ns
ns
ns
1.5
1.0
0.5
0.0
OD 450nm
NC
25mMGlu+0 uM PA
25mMGlu+50 uM PA
25mMGlu+100 uM PA
25mMGlu+200 uM PA
25mMGlu+400 uM PA
25mMGlu+800 uM PA
C
25mMGlu+0uMPA
25mMGlu+50uMPA
25mMGlu+100uMPA
25mMGlu+200uMPA
25mMGlu+400uMPA
25mMGlu+800uMPA
2.5
2.0
1.5
1.0
0.5
0.0
OD 450nm
0
24
48
72
(h)
****
****
*
1.5
1.0
0.5
0.0
OD 450nm
0
24
48
72
Time(h)
