# Supplementary material for: Upregulation of SQSTM1 Regulates Ferroptosis and Oxidative Stress in Müller Cells of the Diabetic Neural Retina by Modulating ACSL4
Source: J Diabetes Res. 2025 Aug 13;2025:1924668. doi: 10.1155/jdr/1924668 (PMC12367365; doi:10.1155/jdr/1924668)
Supplement: Supporting Information — Additional supporting information can be found online in the Supporting Information section. The supporting information for this article can be found online at the following: Figure S1: Immunofluorescence identification of Müller cells. Figure S2: High glucose/palmitic acid (HGP) treatment reduces Müller cell viability. Figure S3: Validation of Sqstm1 knockdown and overexpression efficiency in Müller cells. Table S1: Forward and reverse sequences of each gene analyzed by real-time polymerase chain reaction. [file 1924668.f1.zip › Supplementary table.docx]

**Supplementary Table 1** ｜ **Forward and reverse sequences of each gene analyzed by realtime polymerase chain reaction**

| Primer name | Sequence (5' → 3') |
| --- | --- |
| mGPX4-RT-F | CCTCTGCTGCAAGAGCCTCCC |
| mGPX4-RT-R | CTTATCCAGGCAGACCATGTGC |
| mxCT-RT-F | GCGACATACTCAAGCAGGAGCA |
| mxCT-RT-R | AGTGGTAACCGCTCAGGTGTTG |
| mSQSTM1-RT-F | CCGCCTGACACCTGCCTCTC |
| m SQSTM1-RT-R | TGCCCTCGCTGTTCCCATCC |
| mβ-actin*-*RT-F | CAGCCTTCCTTCTTGGGTATG |
| mβ-actin*-*RT-R | GGCATAGAGGTCTTTACGGATG |
