# Supplementary material for: Upregulation of SQSTM1 Regulates Ferroptosis and Oxidative Stress in Müller Cells of the Diabetic Neural Retina by Modulating ACSL4
Source: J Diabetes Res. 2025 Aug 13;2025:1924668. doi: 10.1155/jdr/1924668 (PMC12367365; doi:10.1155/jdr/1924668)
Supplement: Supporting Information — Additional supporting information can be found online in the Supporting Information section. The supporting information for this article can be found online at the following: Figure S1: Immunofluorescence identification of Müller cells. Figure S2: High glucose/palmitic acid (HGP) treatment reduces Müller cell viability. Figure S3: Validation of Sqstm1 knockdown and overexpression efficiency in Müller cells. Table S1: Forward and reverse sequences of each gene analyzed by real-time polymerase chain reaction. [file 1924668.f1.zip › WB-Figure.pdf]

A

Figure1-M β-actin

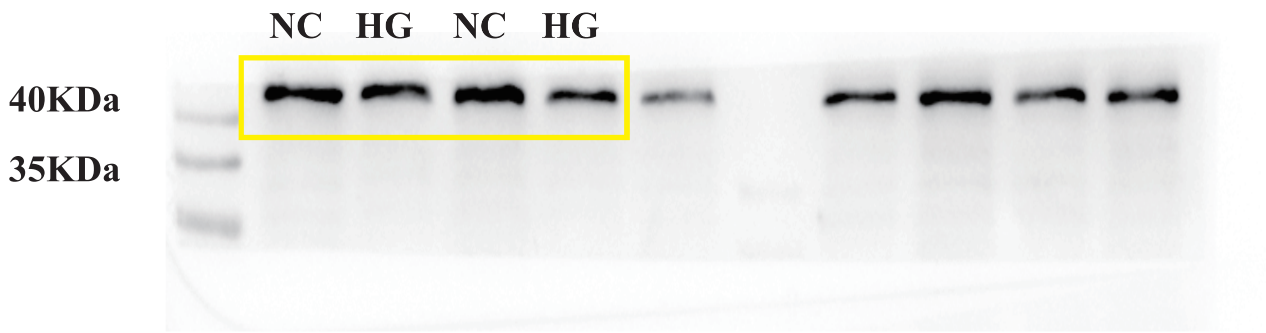

B

Figure1-M xCT

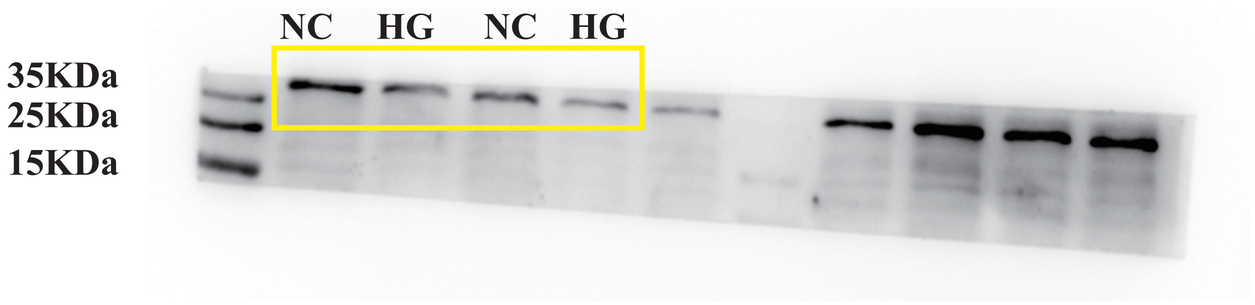

C

Figure1-M GXP4

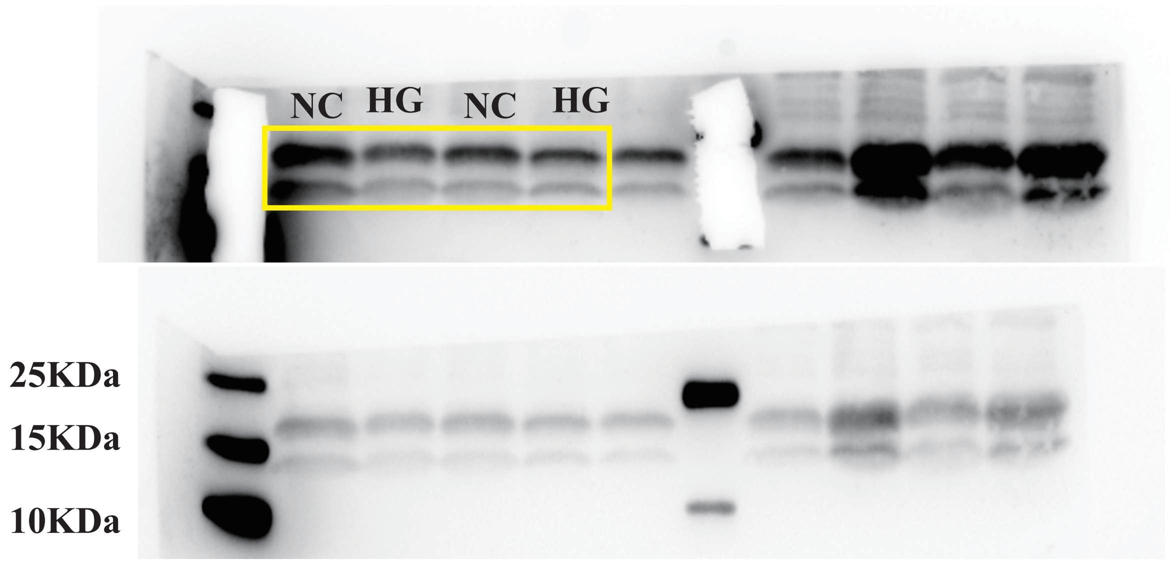

D

Figure1-M

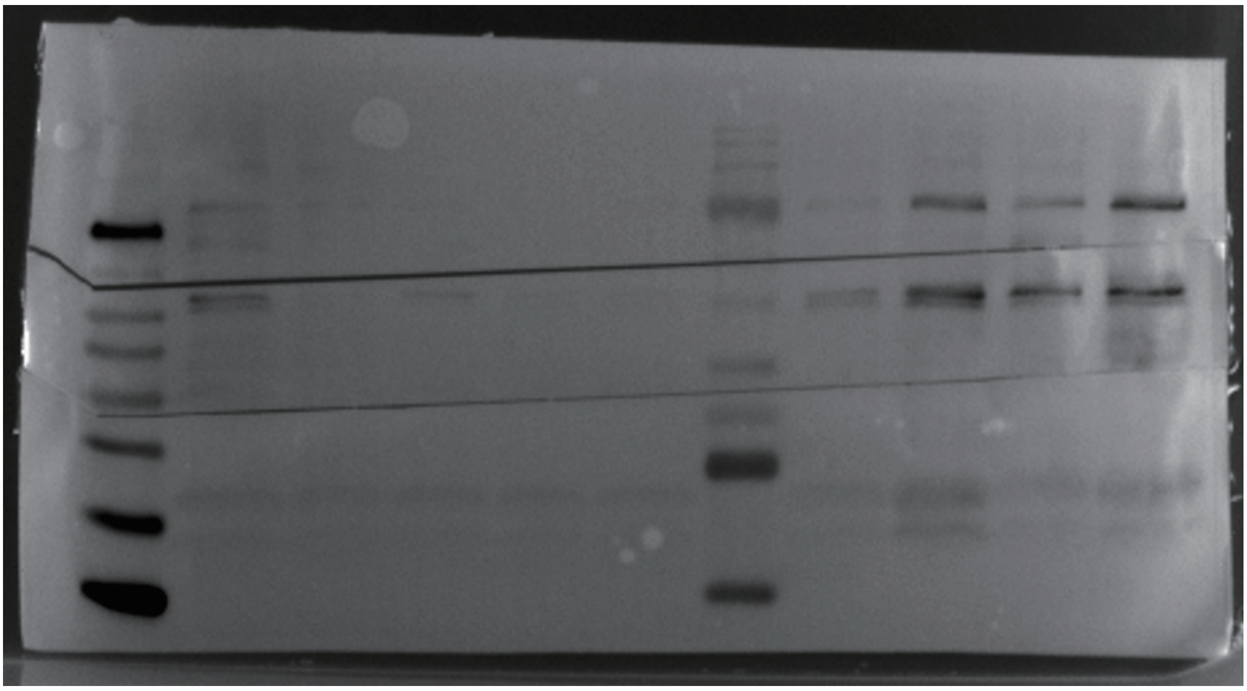

A,B,C and D are from Figure.1-M

A

Figure2-O SQSTM1

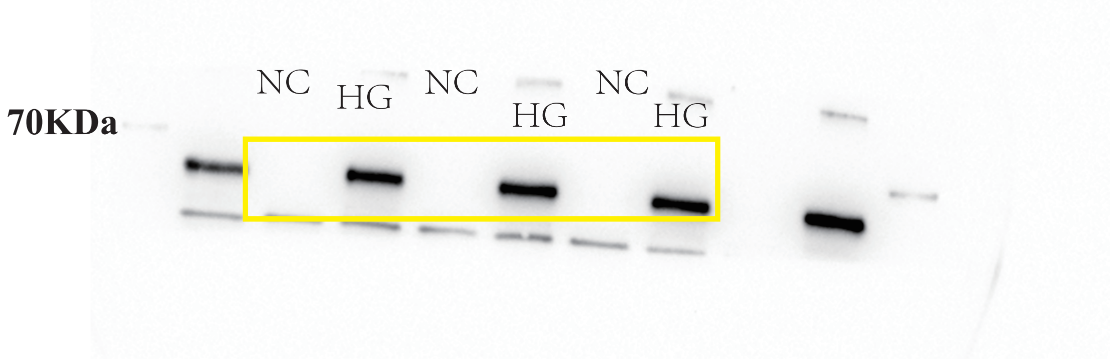

B

Figure2-O β-actin

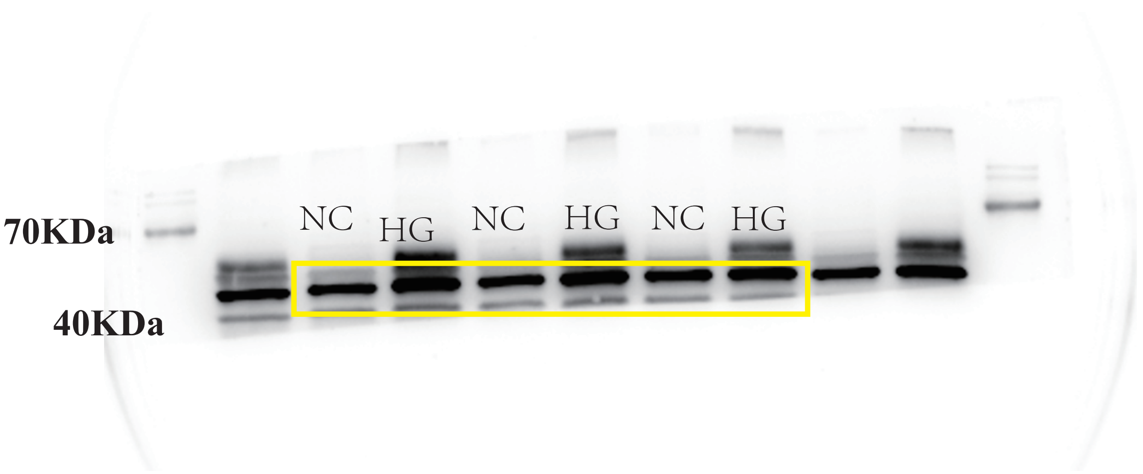

C

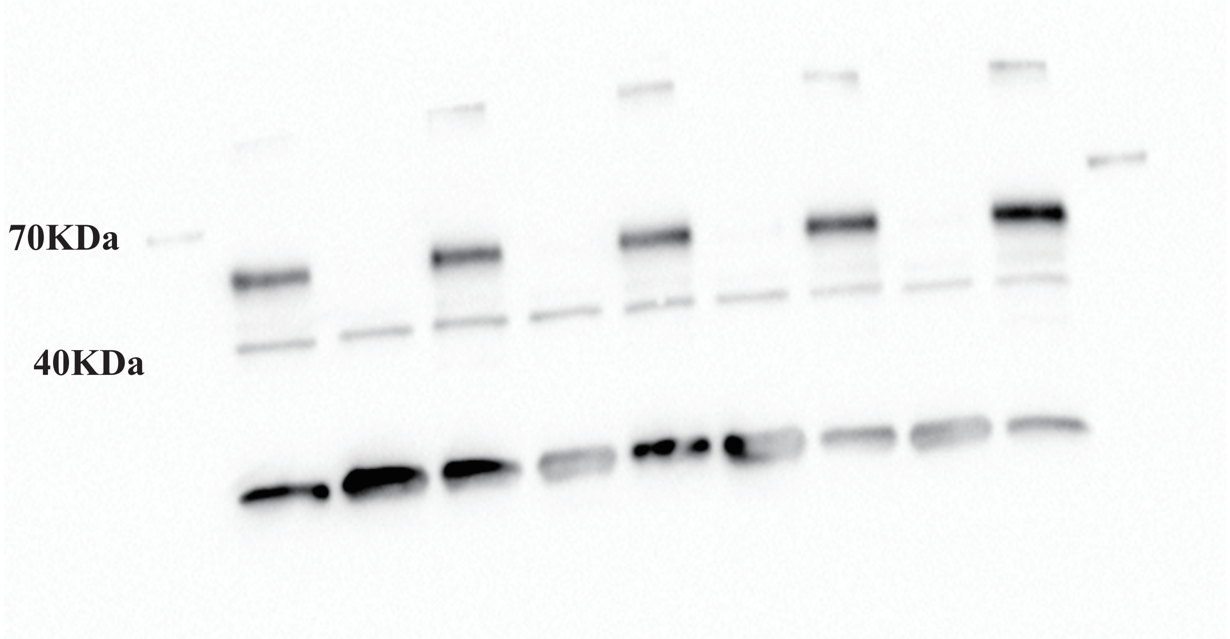

D

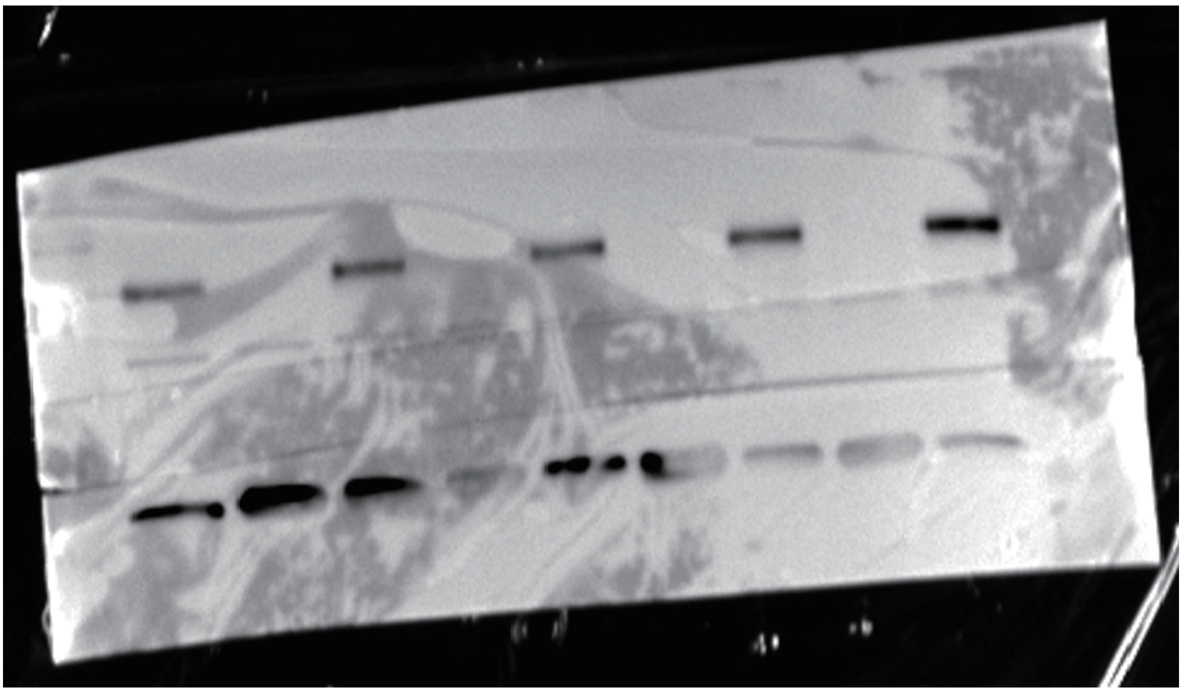

A,B,C and D make up Figure2-O

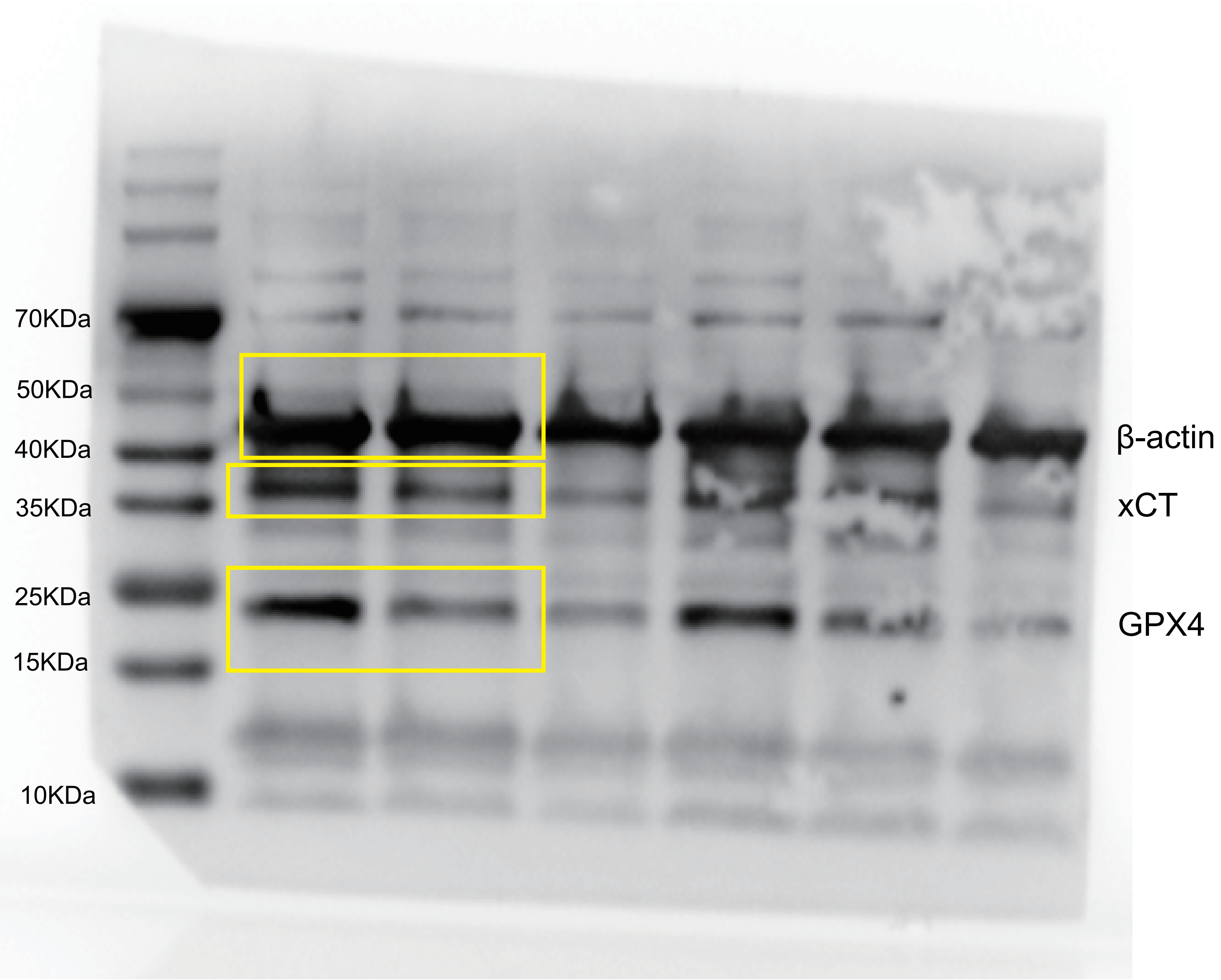

The figure is from Figure4-A

A

Figure4-L SQSTM1

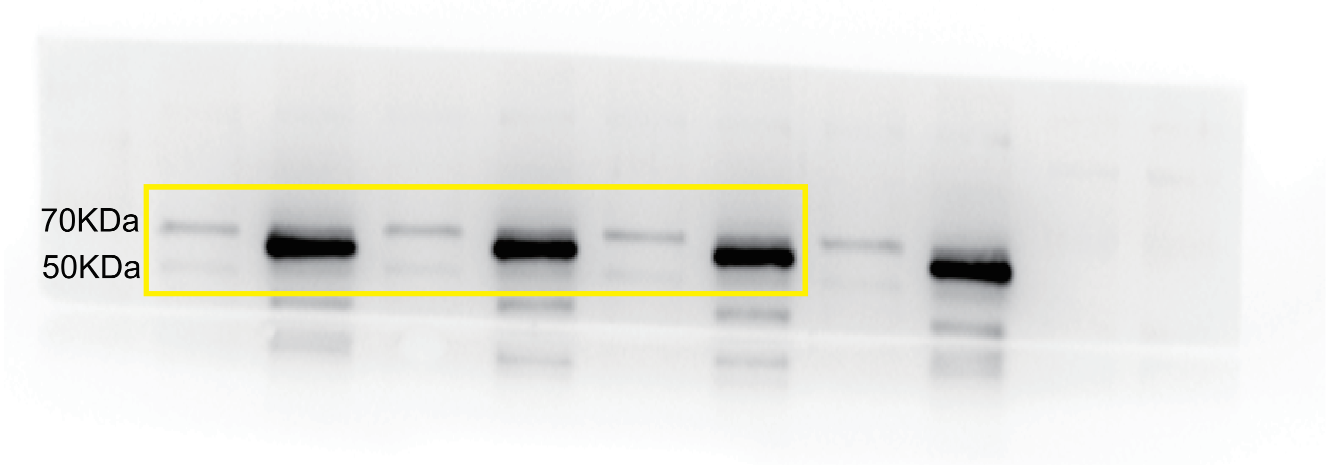

B

Figure4-L β-actin

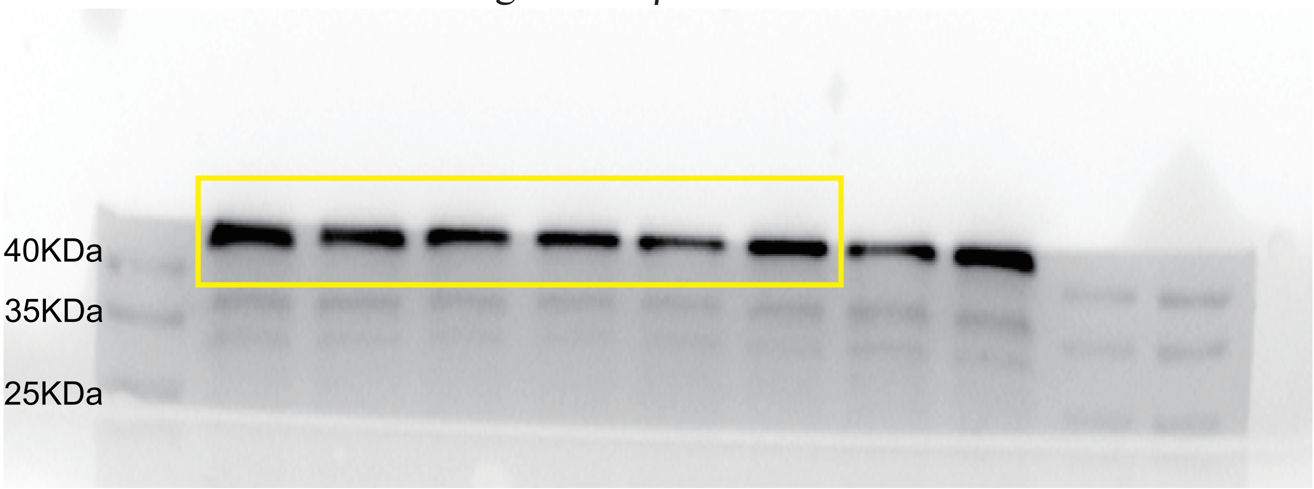

C

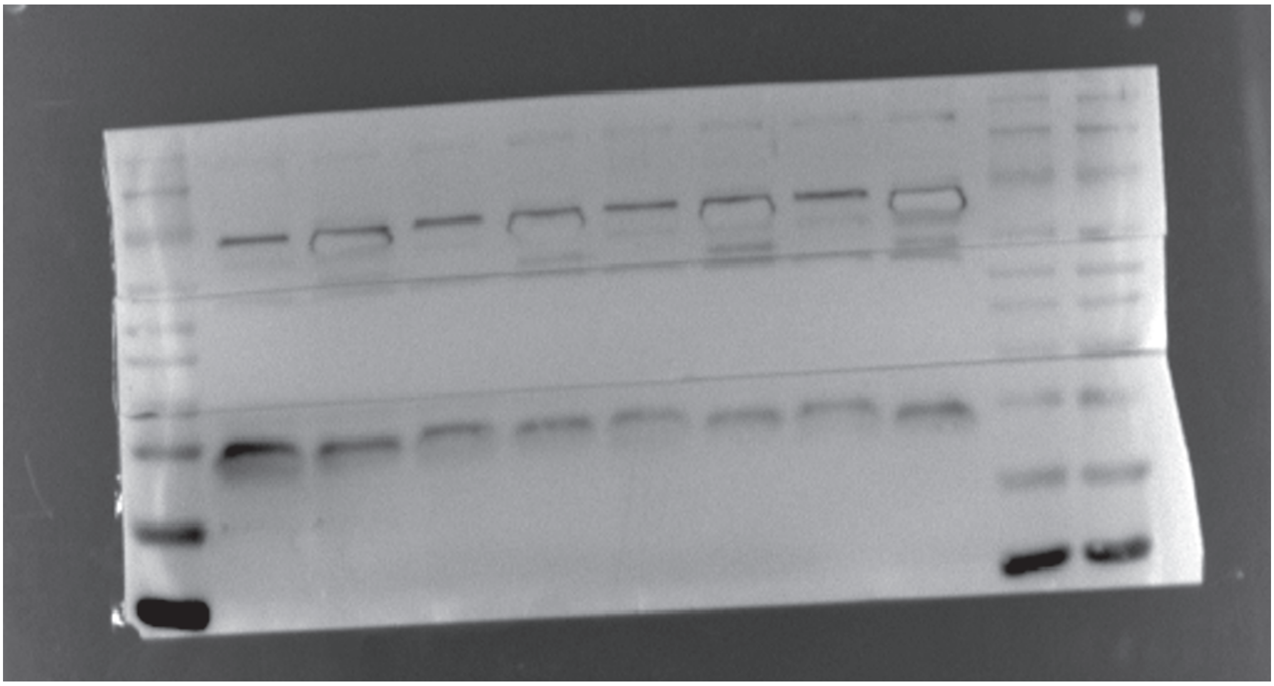

A,B and C D make up Figures4-L

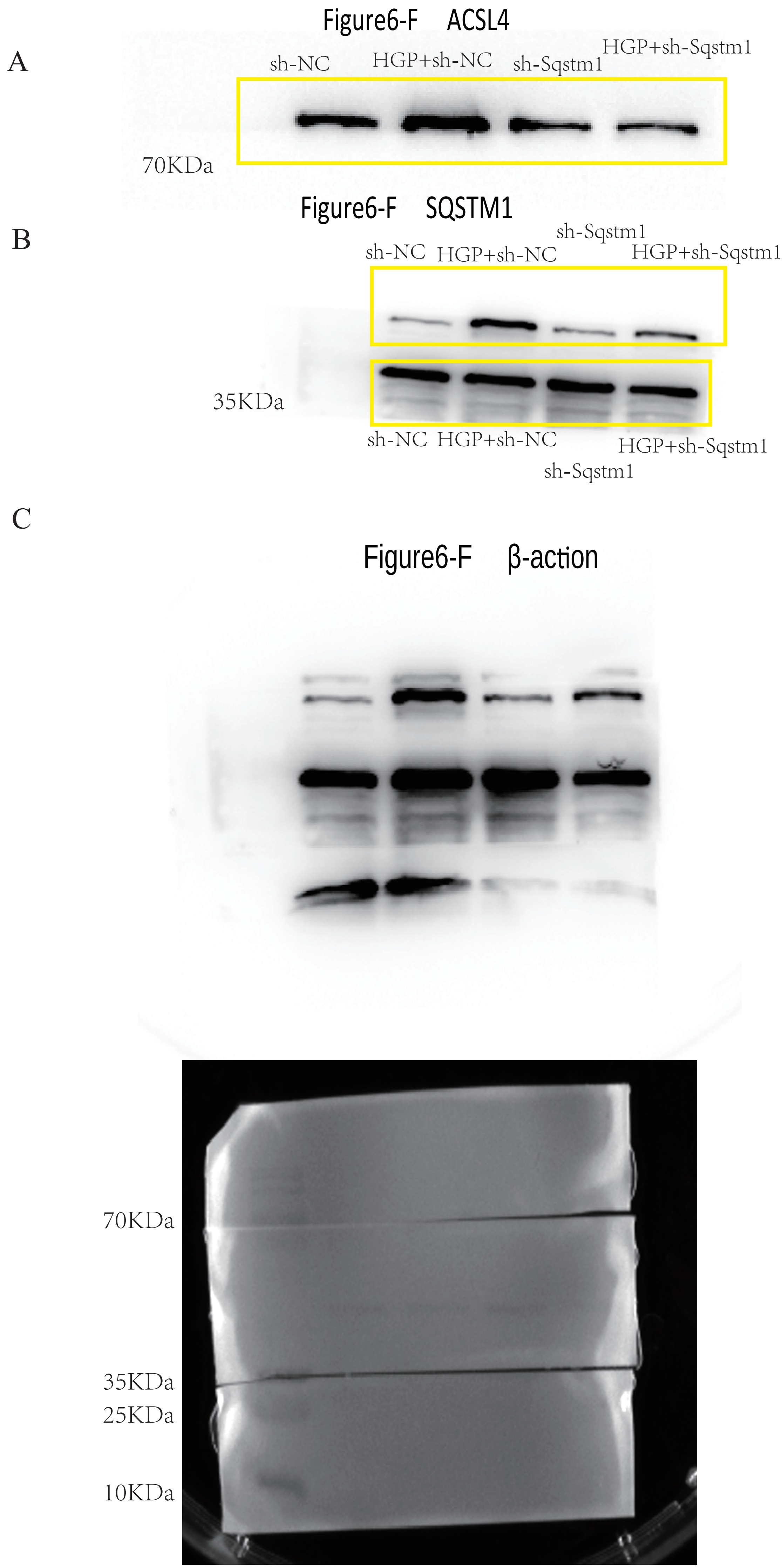

A,B,and C make up Figure6-F

A

Figure6-G ACSL4

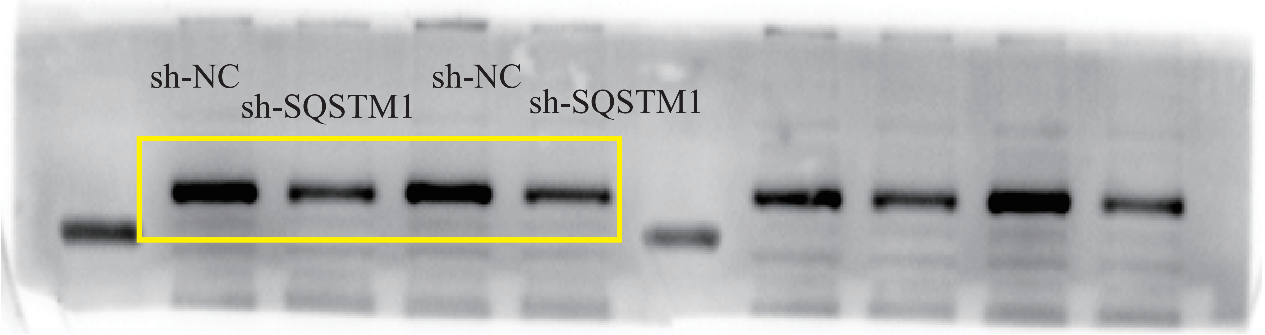

B

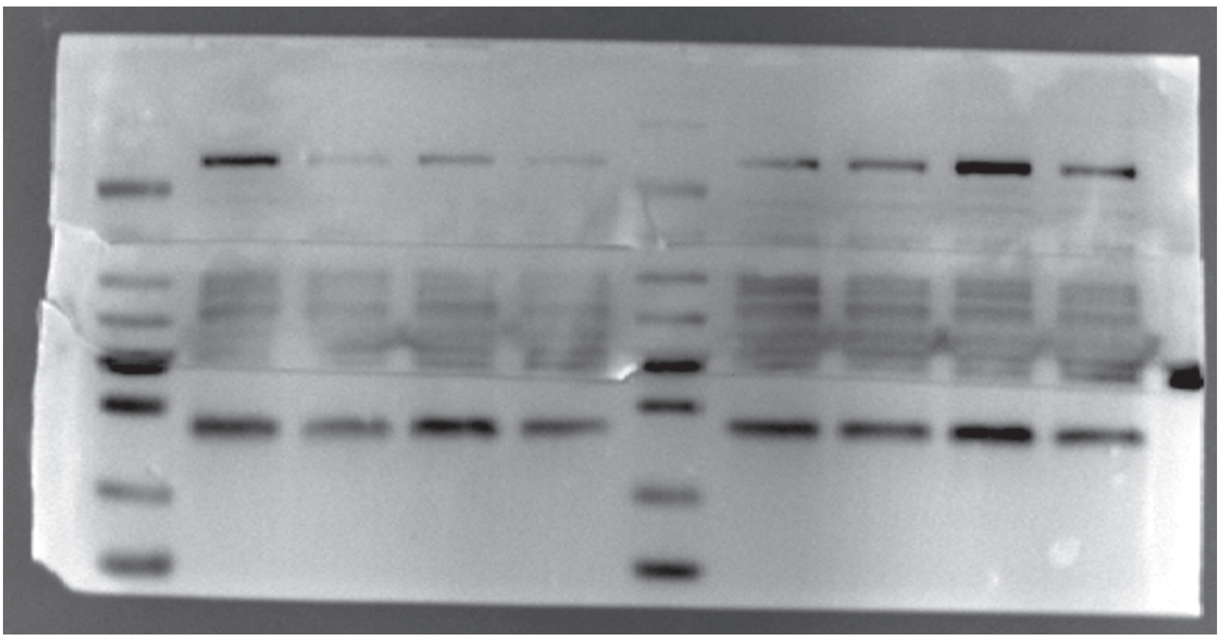

Figure6-G SQSTM1

C

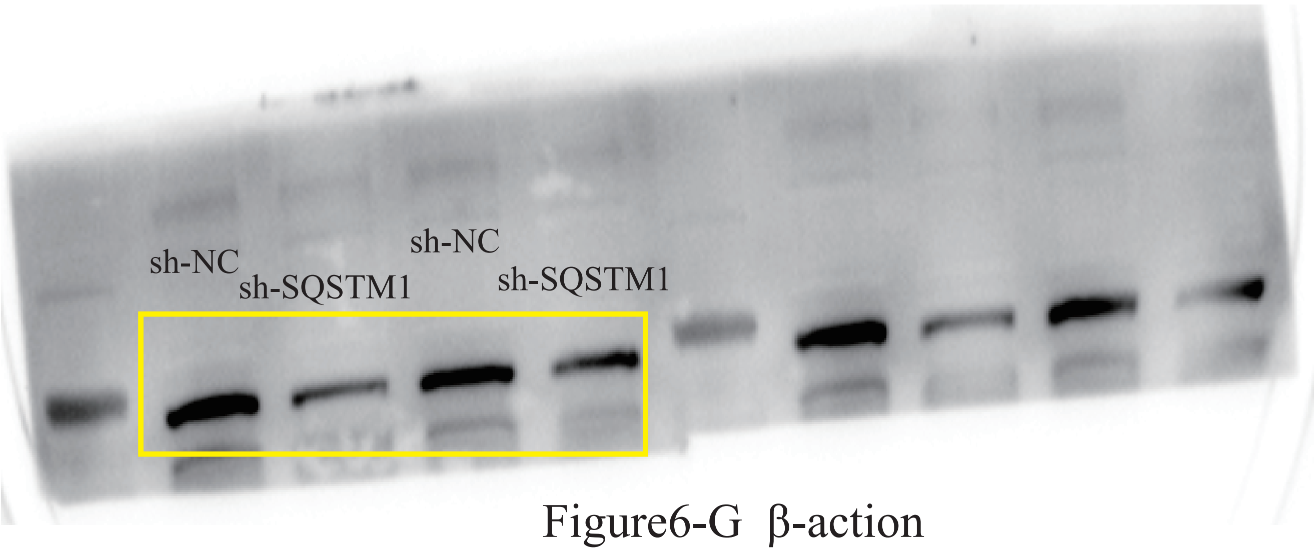

Figure6-G β-action

D

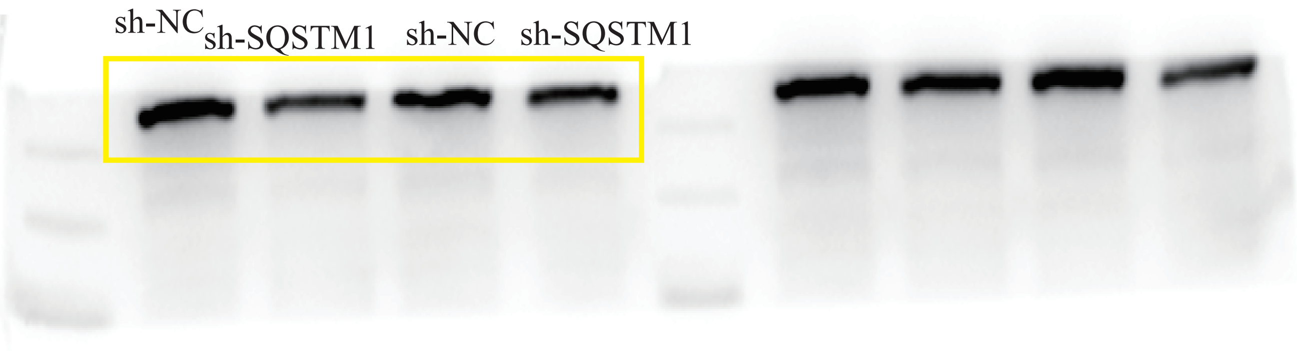

E

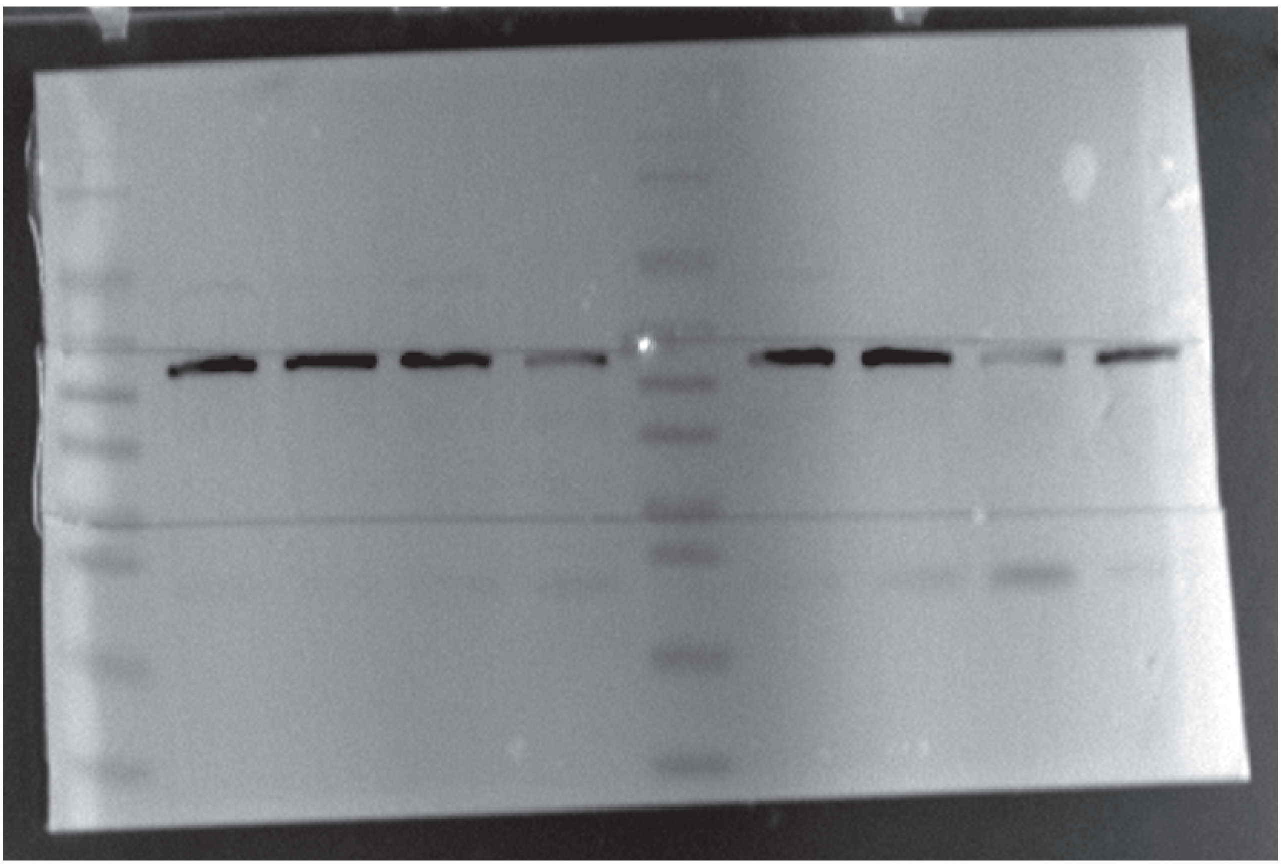

A,B,C,D ,and E make up Figure6-G

A

Figure6-H ACSL4

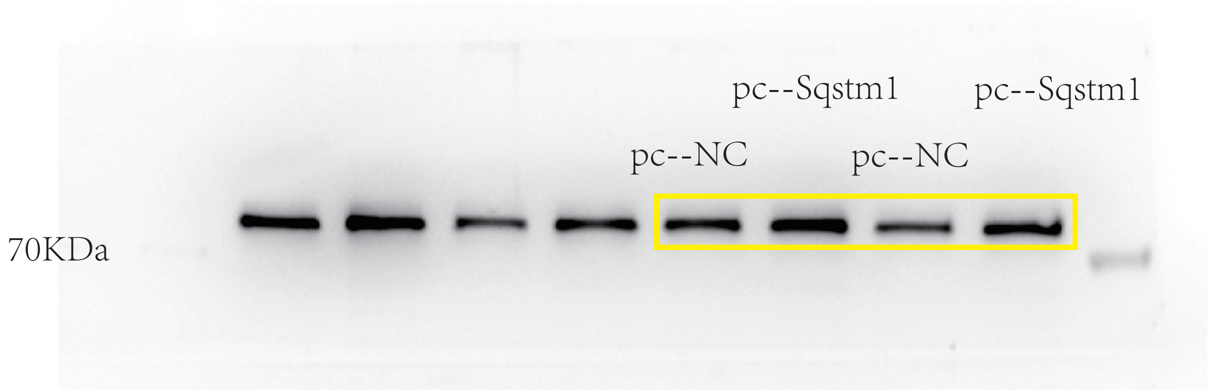

B

Figure6-H Sqstm1

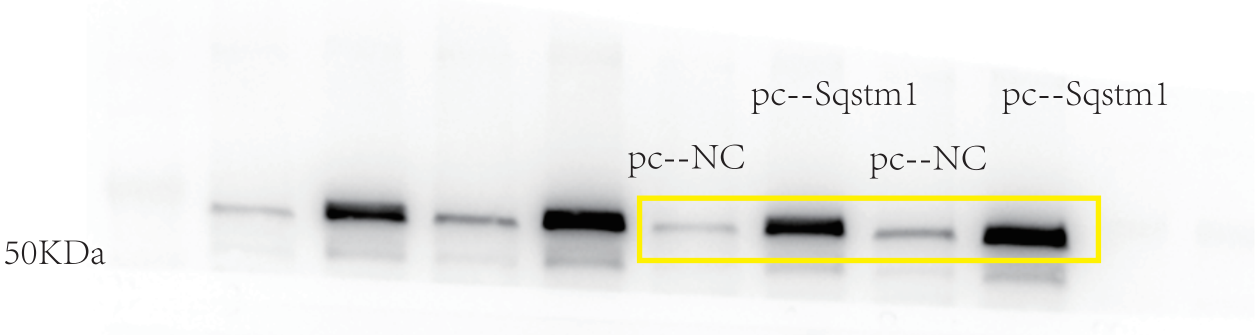

Figure6-H β-actin

C

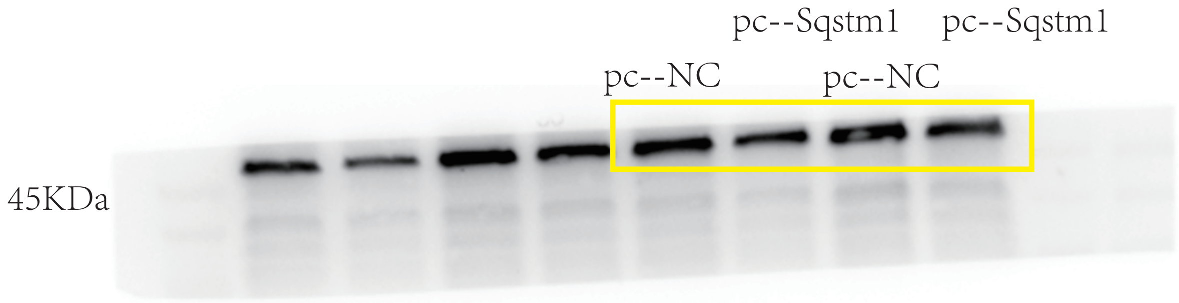

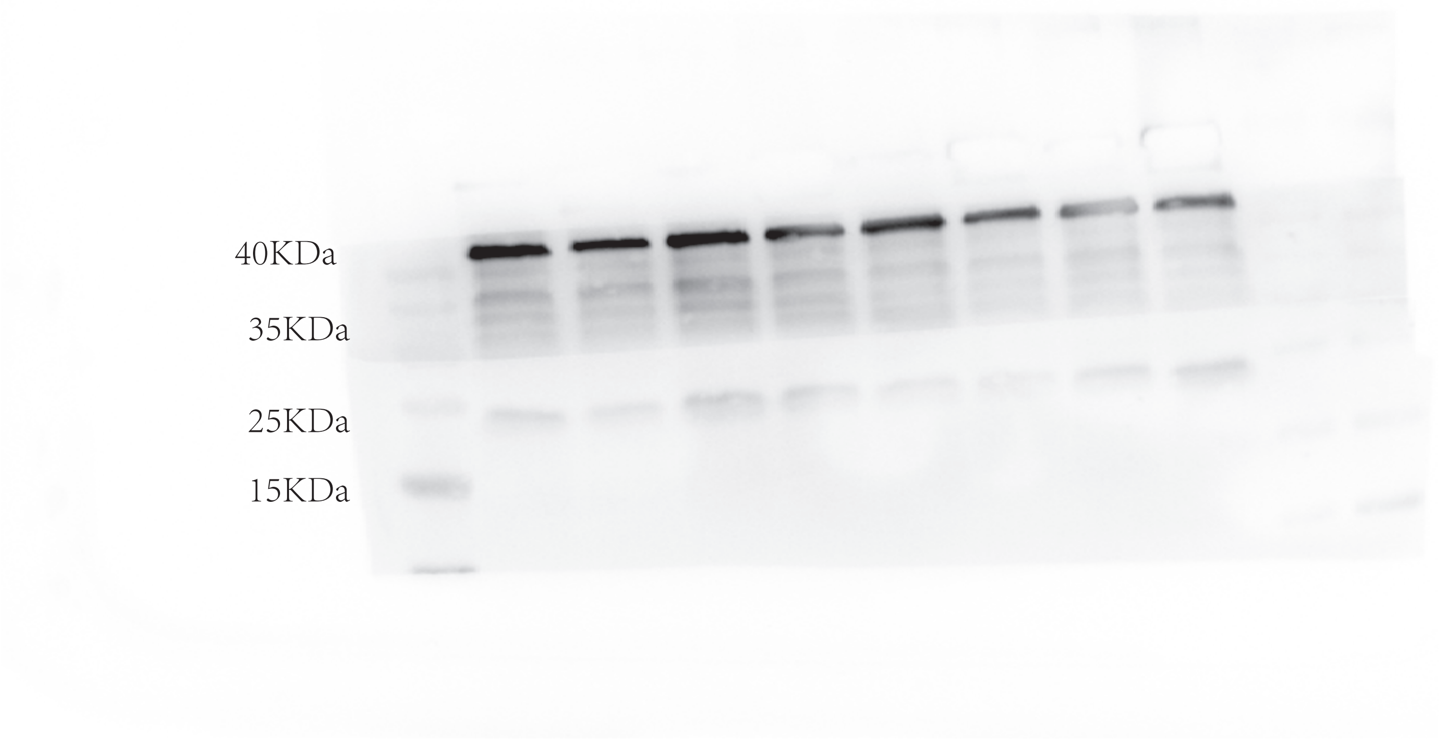

A,B,and C make up Figure6-H

A

FigureS3-A
SQSTM1

70KDa
55KDa

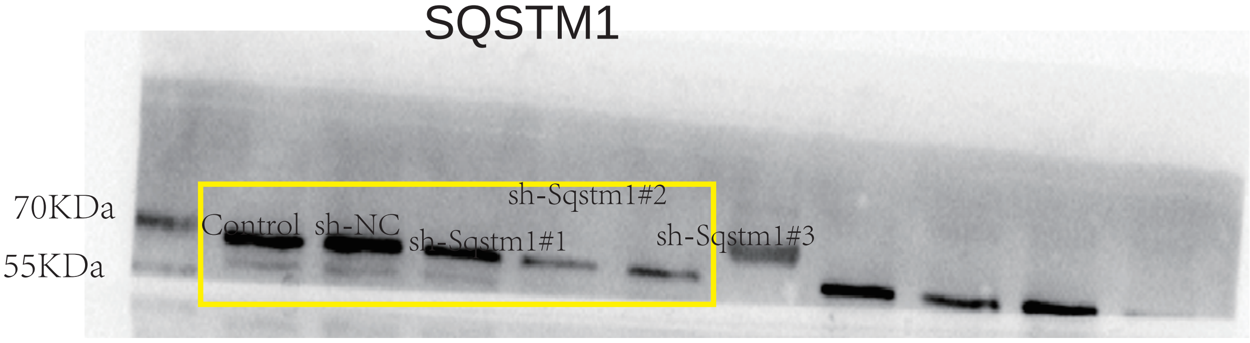

SQSTM1

B

FigureS3-A β-actin

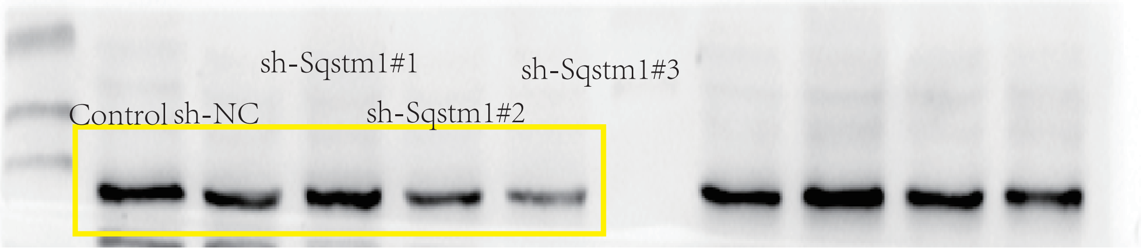

β -actin

C

70KDa
55KDa
40KDa
35KDa
25KDa
15KDa
10KDa

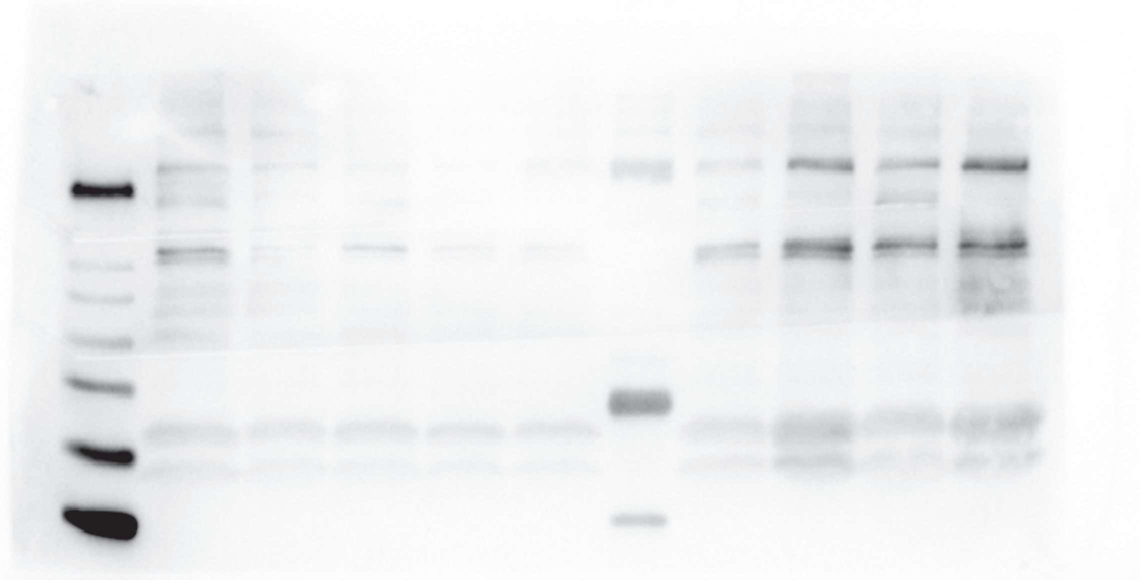

A,B,and C make up Figure3S-A

A

FigureS3-C SQSTM1

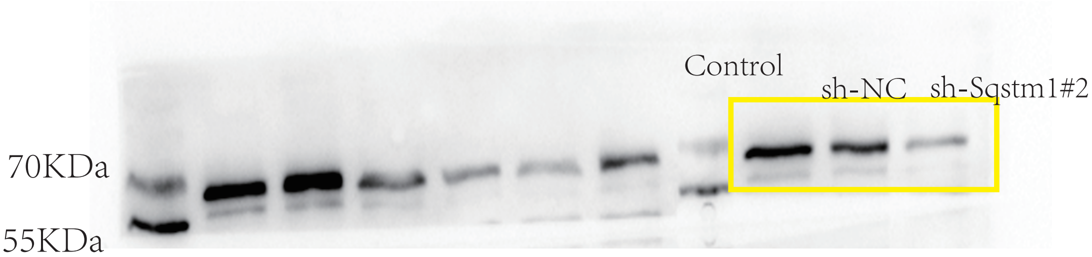

B

FigureS3-C β-actin

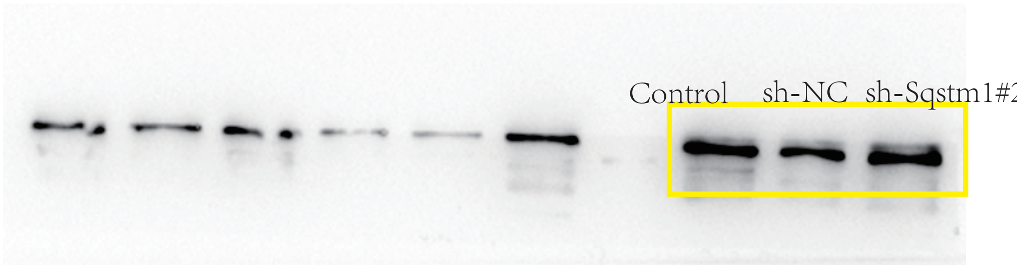

C

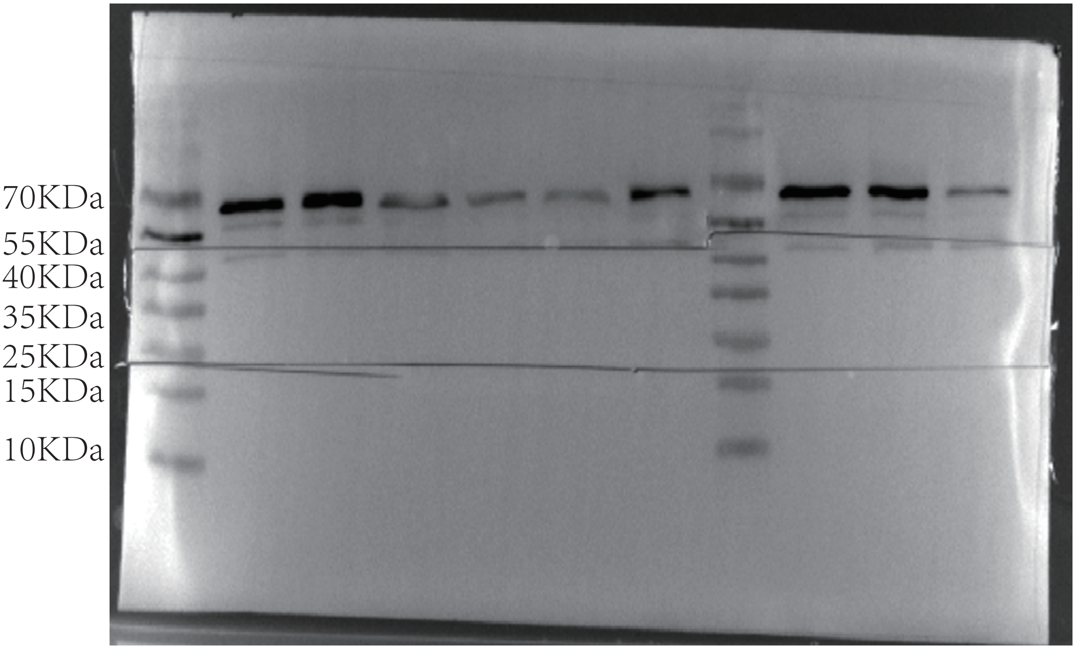

A,B,and C make up FigureS3-C

A

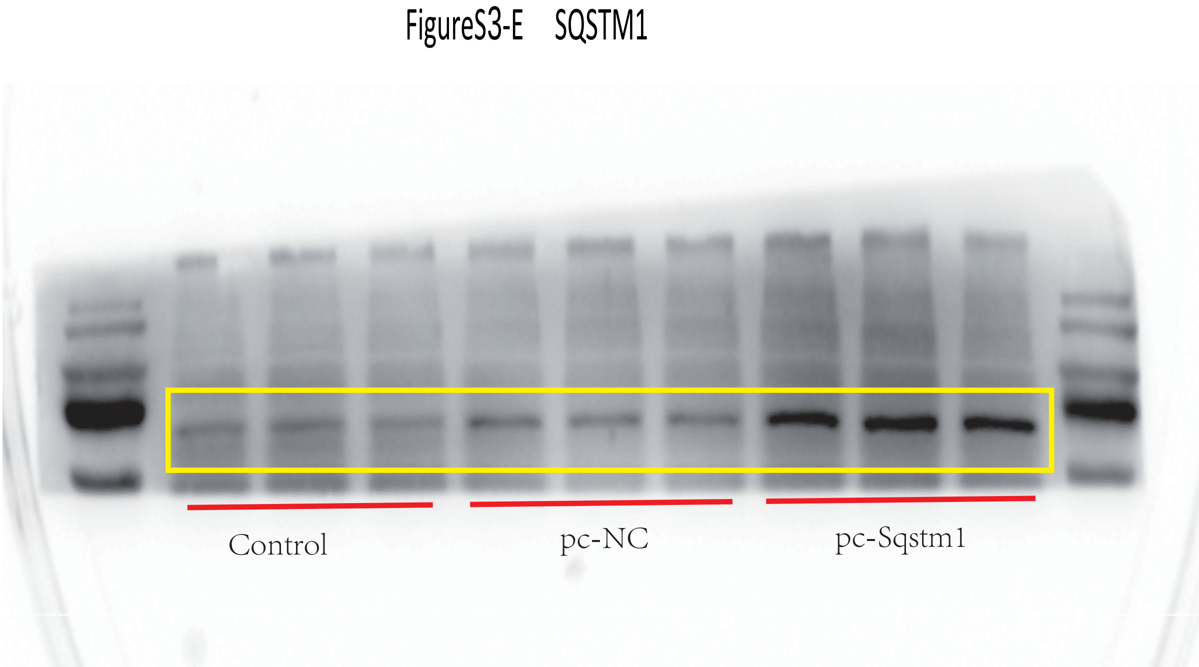

B

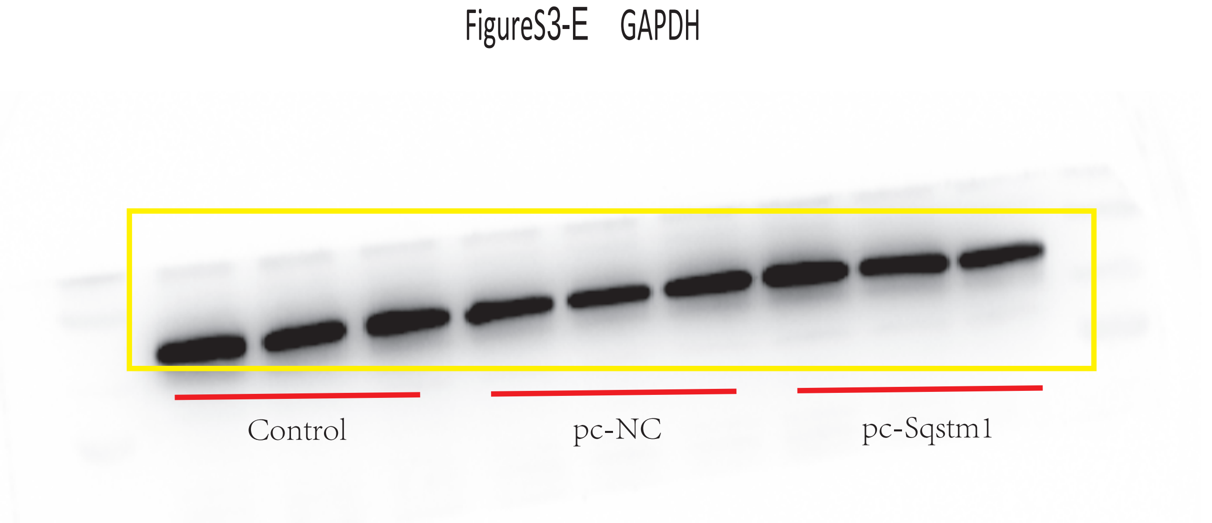

C

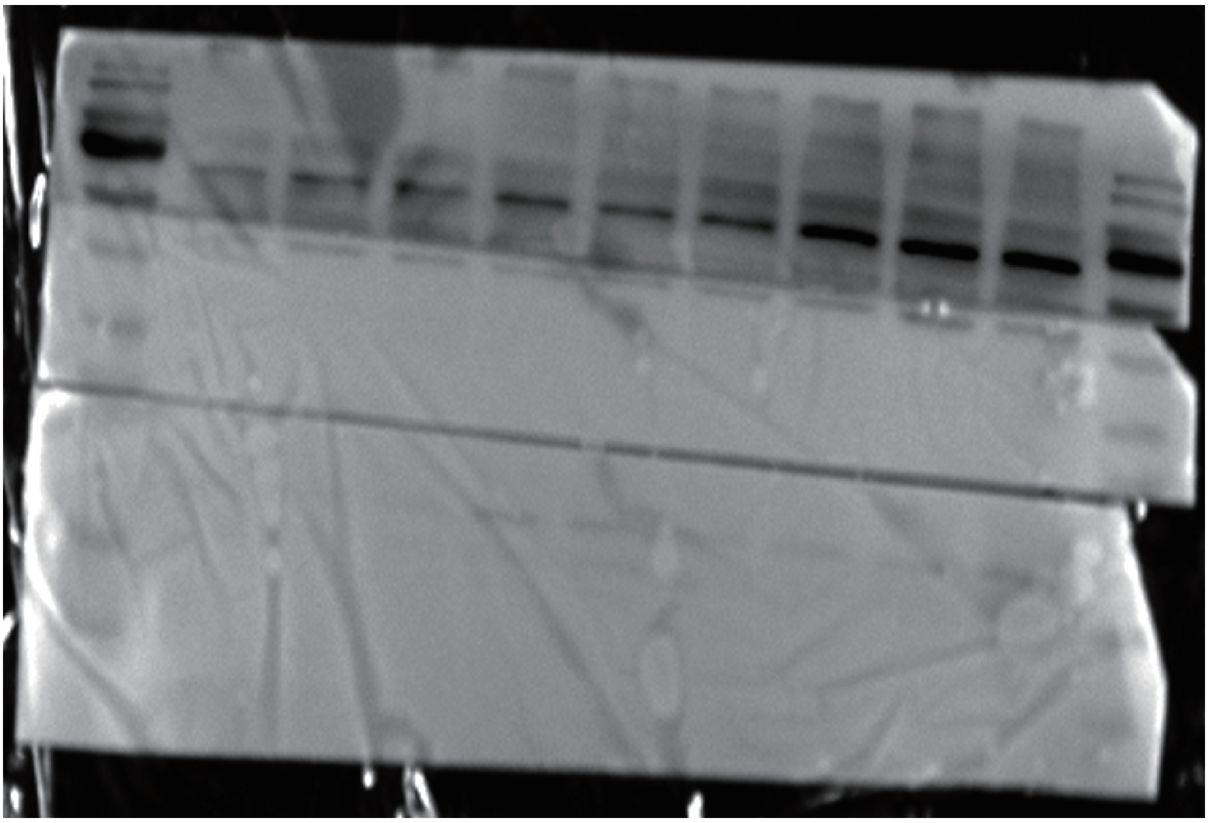

A,B,and C make up FigureS3-E
