# Supplementary figures and images for: Upregulation of SQSTM1 Regulates Ferroptosis and Oxidative Stress in Müller Cells of the Diabetic Neural Retina by Modulating ACSL4
Source: J Diabetes Res. 2025 Aug 13;2025:1924668. doi: 10.1155/jdr/1924668 (PMC12367365; doi:10.1155/jdr/1924668)

A

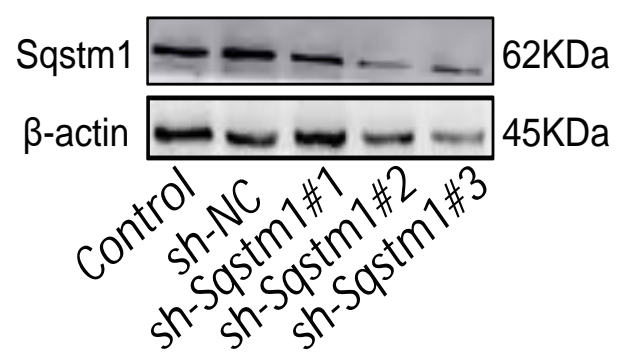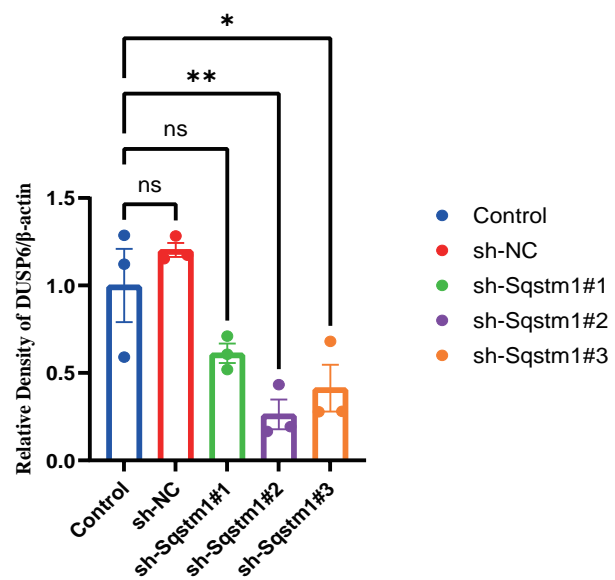

B

sh-NC

sh-Sqstm1#2

FL

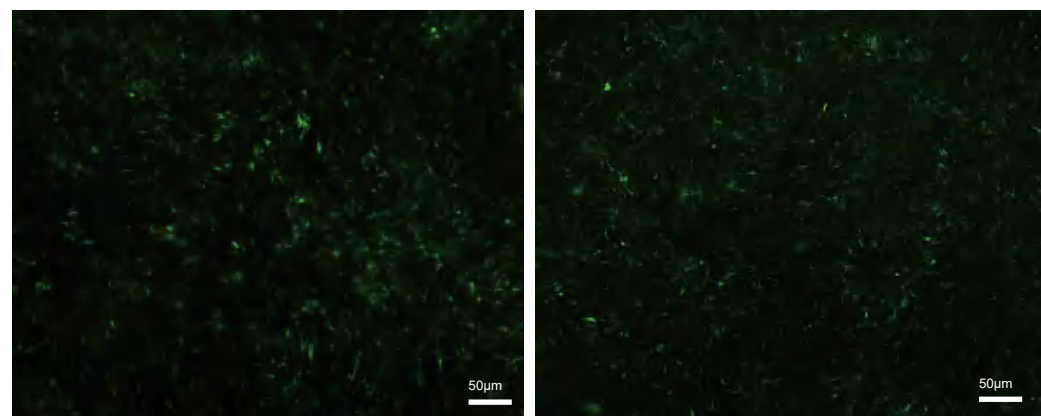

Bright

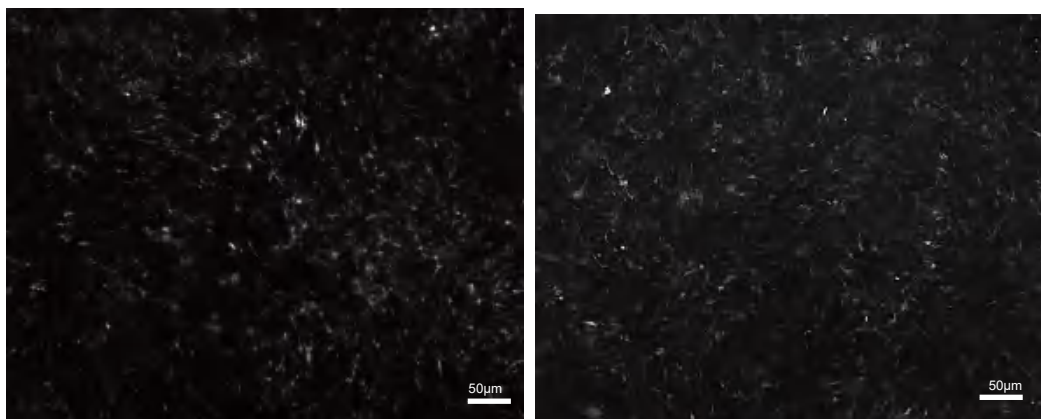

C

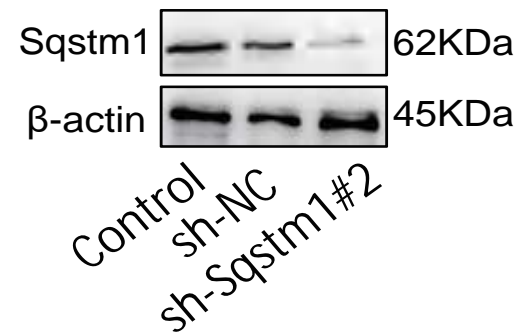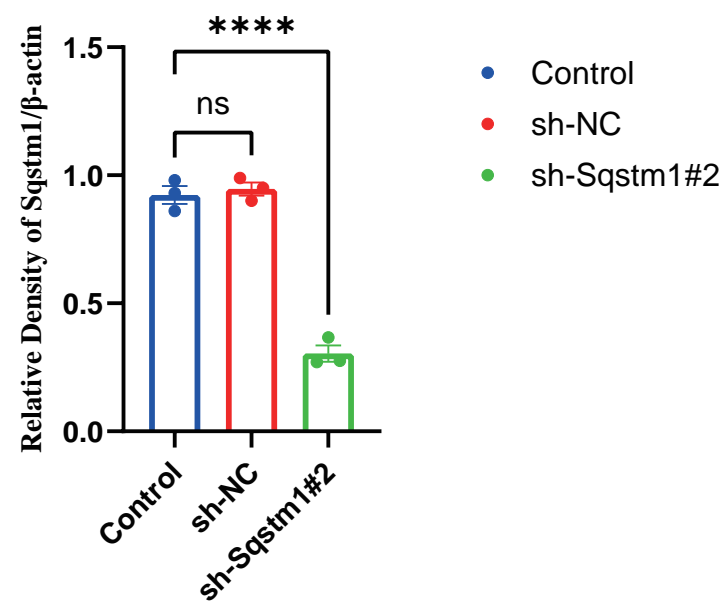

D

PC-NC

PC-Sqstm1

FL

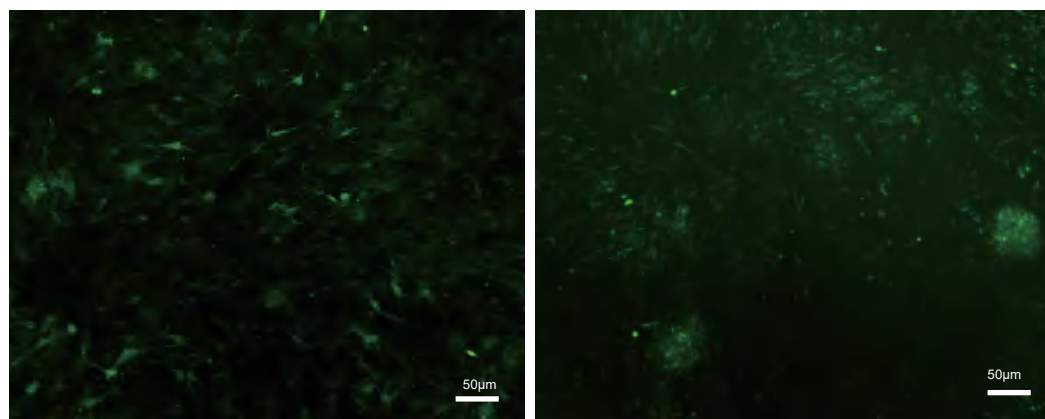

Bright

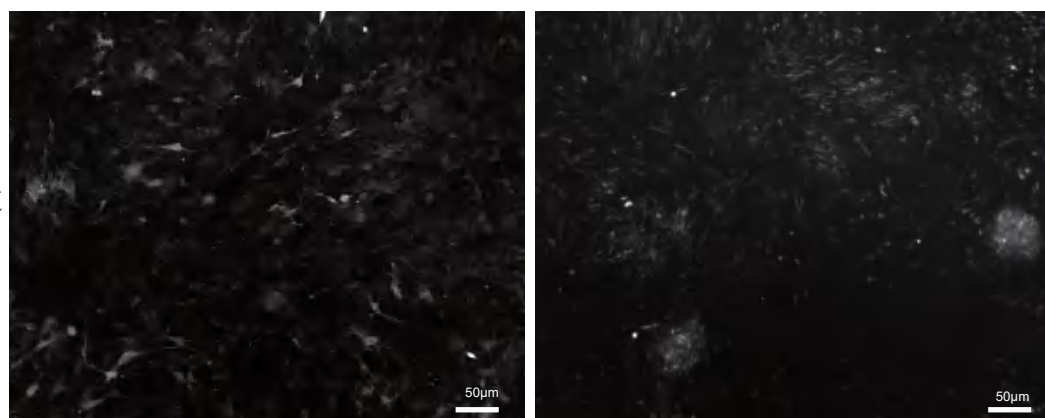

E

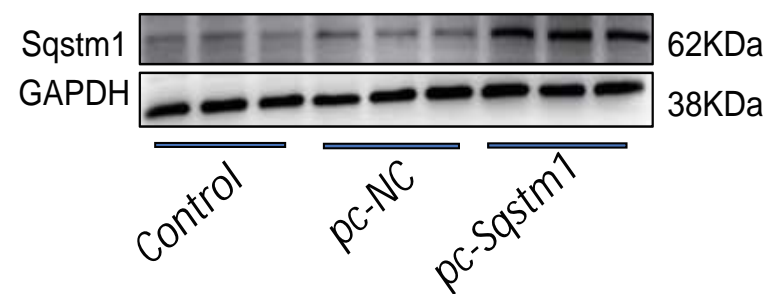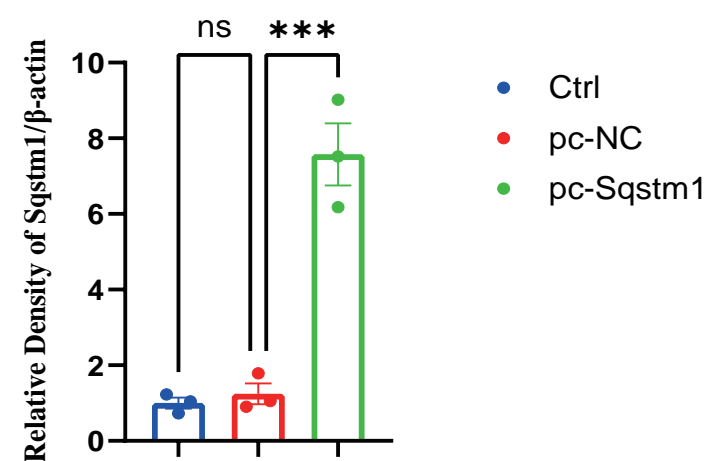

Supplement: Supporting Information — Additional supporting information can be found online in the Supporting Information section. The supporting information for this article can be found online at the following: Figure S1: Immunofluorescence identification of Müller cells. Figure S2: High glucose/palmitic acid (HGP) treatment reduces Müller cell viability. Figure S3: Validation of Sqstm1 knockdown and overexpression efficiency in Müller cells. Table S1: Forward and reverse sequences of each gene analyzed by real-time polymerase chain reaction. [file 1924668.f1.zip › (___)Supplementary Figure3.pdf]

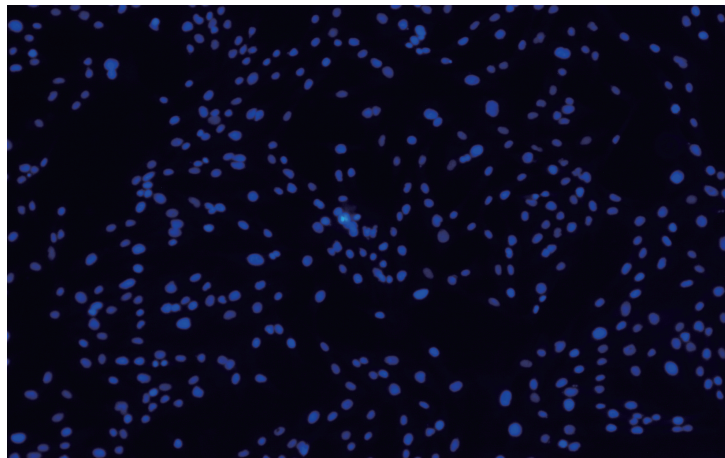

100X-DAPI

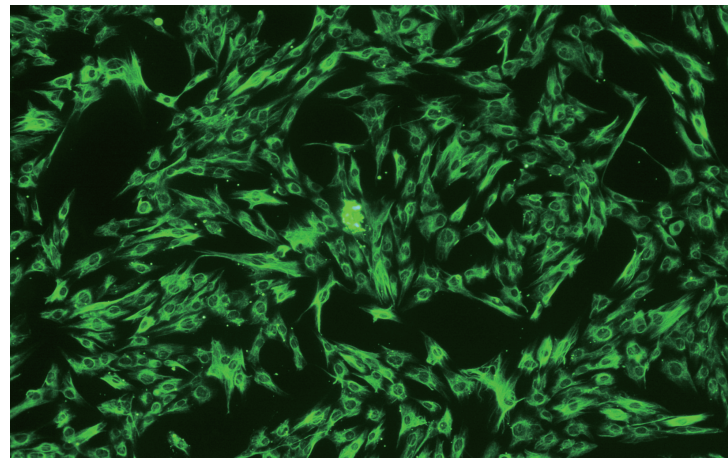

100X-Fuorescence

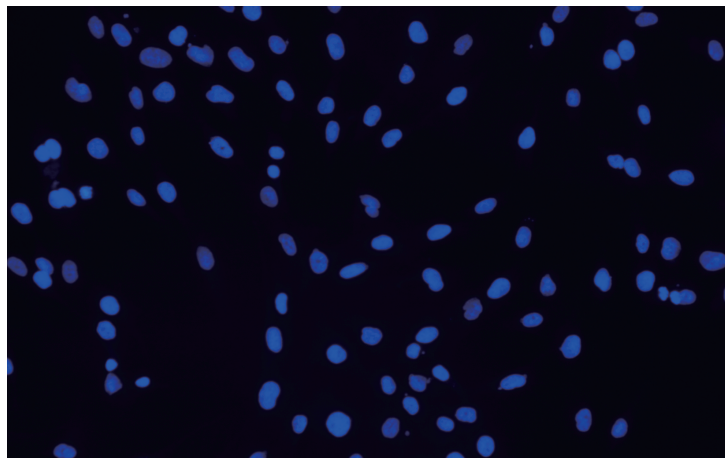

200X-DAPI

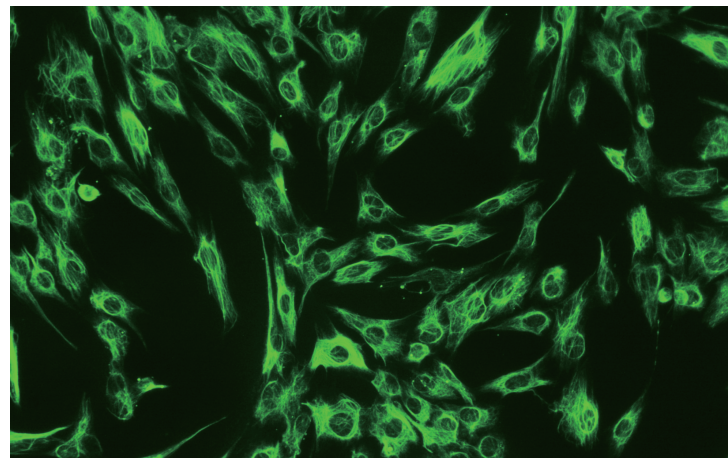

200X-Fuorescence

Supplement: Supporting Information — Additional supporting information can be found online in the Supporting Information section. The supporting information for this article can be found online at the following: Figure S1: Immunofluorescence identification of Müller cells. Figure S2: High glucose/palmitic acid (HGP) treatment reduces Müller cell viability. Figure S3: Validation of Sqstm1 knockdown and overexpression efficiency in Müller cells. Table S1: Forward and reverse sequences of each gene analyzed by real-time polymerase chain reaction. [file 1924668.f1.zip › Supplementary Figure1.pdf]
